# Insights into metabolic osmoadaptation of the ectoines-producer bacterium *Chromohalobacter salexigens* through a high-quality genome scale metabolic model

**DOI:** 10.1186/s12934-017-0852-0

**Published:** 2018-01-09

**Authors:** Francine Piubeli, Manuel Salvador, Montserrat Argandoña, Joaquín J. Nieto, Vicente Bernal, Jose M. Pastor, Manuel Cánovas, Carmen Vargas

**Affiliations:** 1 Department of Microbiology and Parasitology, Faculty of Pharmacy, University of Sevilla, C/Profesor García González 2, 41012 Sevilla, Spain; 2 Department of Biochemistry and Molecular Biology B and Immunology, Faculty of Chemistry, Campus Regional de Excelencia Internacional “Campus Mare Nostrum”, University of Murcia, 30100 Murcia, Spain; 3 Centro de Tecnología de Repsol, REPSOL S.A. Calle Agustín de Betancourt, s/n. 28935, Móstoles, Madrid Spain

**Keywords:** Genome–scale metabolic model, Flux balance analysis, *Chromohalobacter salexigens*, Metabolic osmoadaptation

## Abstract

**Background:**

The halophilic bacterium *Chromohalobacter salexigens* is a natural producer of ectoines, compatible solutes with current and potential biotechnological applications. As production of ectoines is an osmoregulated process that draws away TCA intermediates, bacterial metabolism needs to be adapted to cope with salinity changes. To explore and use *C. salexigens* as cell factory for ectoine(s) production, a comprehensive knowledge at the systems level of its metabolism is essential. For this purpose, the construction of a robust and high-quality genome-based metabolic model of *C. salexigens* was approached.

**Results:**

We generated and validated a high quality genome-based *C. salexigens* metabolic model (*i*FP764). This comprised an exhaustive reconstruction process based on experimental information, analysis of genome sequence, manual re-annotation of metabolic genes, and in-depth refinement. The model included three compartments (periplasmic, cytoplasmic and external medium), and two salinity-specific biomass compositions, partially based on experimental results from *C. salexigens.* Using previous metabolic data as constraints, the metabolic model allowed us to simulate and analyse the metabolic osmoadaptation of *C. salexigens* under conditions for low and high production of ectoines. The *i*FP764 model was able to reproduce the major metabolic features of *C. salexigens*. Flux Balance Analysis (FBA) and Monte Carlo Random sampling analysis showed salinity-specific essential metabolic genes and different distribution of fluxes and variation in the patterns of correlation of reaction sets belonging to central C and N metabolism, in response to salinity. Some of them were related to bioenergetics or production of reducing equivalents, and probably related to demand for ectoines. Ectoines metabolic reactions were distributed according to its correlation in four modules. Interestingly, the four modules were independent both at low and high salinity conditions, as they did not correlate to each other, and they were not correlated with other subsystems.

**Conclusions:**

Our validated model is one of the most complete curated networks of halophilic bacteria. It is a powerful tool to simulate and explore *C. salexigens* metabolism at low and high salinity conditions, driving to low and high production of ectoines. In addition, it can be useful to optimize the metabolism of other halophilic bacteria for metabolite production.

**Electronic supplementary material:**

The online version of this article (10.1186/s12934-017-0852-0) contains supplementary material, which is available to authorized users.

## Background

*Chromohalobacter salexigens* is a moderately halophilic gamma-proteobacterium of the family *Halomonadaceae*, phylogenetically related to the genus *Halomonas* [1]. It displays one of the broadest known growth salinity ranges [2], and is considered as a biological model to study prokaryotic osmoadaptation. *C. salexigens* has been proposed, among other natural producers, as a cell factory for the production of the biostabilizing compatible solutes ectoine and hydroxyectoine [3, 4]. The microorganism has several advantages as a cell factory: (i) it is easily cultured by using conventional media, (ii) it has broad metabolic versatility being capable of using different carbon sources [1], (iii) a high diversity of genetic tools are available for its study [5], and (iv) its genome has been sequenced [6].

To further explore and use *C. salexigens* as cell factory for ectoine(s), a comprehensive understanding of its metabolism is essential. In *C. salexigens*, production of ectoines is an osmoregulated process that burdens central metabolic routes by quantitatively drawing TCA cycle intermediaries. The ectoine biosynthetic pathway starts with the phosphorylation of L-aspartate and consumes oxaloacetate (OAA) and acetyl-CoA, the latter of which is produced by oxidative decarboxylation of pyruvate by pyruvate dehydrogenase. This use of intermediaries of the TCA cycle is of special relevance, since active anaplerotic pathways are needed in order to replenish the cycle [7]. Consequently, central metabolism is adapted to the requirements of this biosynthetic route.

Genome-scale metabolic models (GEMs) are currently the only approach that enables the modeling and global analysis of an organism’s metabolic and transport network by a global analysis [8]. A genome-wide constraint-based model consists of a stoichiometric reconstruction of all reactions known to act in the metabolism of the organism, along with an accompanying set of constraints on the fluxes of each reaction in the system [9]. A major advantage of this approach is that the model does not require knowledge on the kinetics of the reactions. These models define the organism’s global metabolic space, network structural properties, potential flux distribution, and provide a framework with which to navigate through the metabolic wiring of the cell [10, 11]. Through various analysis techniques, constraint-based models can help to predict cellular phenotypes, given particular environmental conditions. Constraint-based analysis techniques (i.e. flux-balance analysis [FBA]), have been instrumental in elucidating metabolic features in a variety of organisms [9] and so far have been used for certain biotechnology endeavors [12, 13].

Here, we describe a highly detailed and curated metabolic reconstruction of *Chromohalobacter salexigens DSM 3043*, one of the mayor ectoine(s) natural producers. The reconstruction was built using the COBRA approach [10, 14] and validated using flux balance analysis [9]. The in silico metabolic network was further evaluated by comparing predicted growth rate capacities in different carbon sources. The model was used to gain insight into the physiology of *C. salexigens* metabolic stress response at conditions of low and high ectoines production. In addition to optimize *C. salexigens* strains for ectoines production, *i*FP764 may become an important tool to understand the metabolism of halophilic microorganisms, and may serve as a platform to test metabolic capabilities of halophilic bacteria at low and high salinity.

## Methods

### Metabolic reconstruction and refinement

The metabolic reconstruction of *C. salexigens* was generated by using a bottom-up approach. This was based on experimental information reported in previous studies, analysis of genome sequence and manual re-annotation of metabolic genes. An initial core model was built up based on previous studies on metabolic osmoadaptation and accumulation of compatible solutes by *C. salexigens,* as well as a thorough literature revision. For this purpose*, g*enes and reactions for the synthesis and degradation of compatible solutes and their connection with the central metabolism, as well as the main C and N pathways, were compiled. Sequentially, this core was expanded with all necessary routes for cell growth. Additionally, gap filling was performed to connect routes and to complete the model. We made sure that main pathways involved in salinity dependent-metabolic adaptations were included in the reconstruction. Thus proteomic and transcriptomic data from experiments performed at low and high salinity (0.6 M and 2.5 M of NaCl respectively) (unpublished results), and previous metabolic data from our laboratory [7], were considered. All pathways, reactions, metabolites, and genes included in the reconstruction were manually inspected. Orthologous sequences of all enzymes of interest were identified and compared using BLAST [15], analyses of domains (Conserved Domain Database [16], and SMART [17]) and Genomic context (STRING [18], and ARCHAEA [19]). Moreover, phylogenetic analyses (ClustalW [20] and MEGA 6 [21] were carried out to correctly assign different isoenzymes to their specific reactions. Reaction directionalities were inspected, and appropriate changes were made based on the BRENDA database [22]. Reactions without any gene association were added based on evidence from the literature, presence in databases, or the need to fill functional gaps to achieve a functional network (as in the case of putative transporters). Exchange reactions were also included, as they are reactions that represent the supply to, or removal of, metabolites from the extra-organism “space” [23]. The reaction rate through the non-growth-associated ATP maintenance reaction (ATPM) was set to 7.6 mmol g^−1^ (dry weight) h^−1^ to account for upkeep. Growth-associated ATP maintenance was included in the two-biomass equations used. Since both equations differ in overall macromolecular composition, the energy cost for these macromolecules synthesis differed concordantly. Thus, we used the Unknown Growth-associated ATP maintenance of 36.94 mmol g^−1^ calculated by Feist et al. [28] and followed their procedure to calculate the use of ATP per mmol of protein, DNA and RNA polymerization at low and high salinity (23.04 and 8.79 mmol g^−1^ respectively). In that way, the Growth-associated ATP maintenance was set to 45.73 mmol g^−1^ (dry weight) at high salinity and 59.98 mmol g^−1^ (dry weight) at low salinity. The molecular formulae and charges of the metabolites in the model were determined assuming a pH of 7.2. The structure and the name of reaction and metabolites were assigned by following an established protocol [23]. All data bases and bioinformatics programs used are listed in Table 1.

**Table 1:** Database sources used for *C. salexigens i*FP764 metabolic reconstruction

Name	Direction
Archaea	http://archaea.u-psud.fr/absynte/
BioCyc	http://biocyc.org/
BioMet ToolBox 2.0	http://biomet-toolbox.org/index.php?page=about
Blast	http://blast.ncbi.nlm.nih.gov/Blast.cgi
Brenda	http://www.brenda-enzymes.org/
ClustalW	http://www.ebi.ac.uk/Tools/msa/clustalw2/
Conserved Domain Database	http://www.ncbi.nlm.nih.gov/cdd
EcoCyc	http://ecocyc.org/
GeneOntology	http://geneontology.org/
Kegg	http://www.genome.jp/kegg/
Mega	http://www.megasoftware.net/
MicrobesOnline	http://www.microbesonline.org/
NCBI	http://www.ncbi.nlm.nih.gov/
Pathway-tools	http://brg.ai.sri.com/ptools/
Smart	http://smart.embl-heidelberg.de/
String	http://string-db.org
TCDB	http://www.tcdb.org/
TransportDB	http://www.membranetransport.org/index.html

#### Formulation of biomass reaction

Given the metabolic adaptation to the salinity of *C. salexigens* [7], the biomass composition defined in the model was adapted to simulate high and low salinity conditions. For that purpose, two different biomass reaction compositions (BIO) were estimated: “BIO_H” to simulate high salinity (2.5 M NaCl), and “BIO_L” to simulate low salinity (0.6 M NaCl). According to Thiele and Palsson [23], the detailed biomass composition needs to be experimentally determined. However, in some occasions, it may not be possible to obtain a detailed biomass composition of the organism of interest. In this case, the main alternative strategy is the utilization of the experimentally determined biomass composition of a non-related microorganism (i.e. *Escherichia coli*) [23]. A second, more accurate, strategy is to partially reformulate an experimentally-determined biomass composition by estimating the relative fraction of the biomass metabolites of the organism of interest that can be experimentally calculated. In this work, both biomass compositions were obtained by partially reformulating *Escherichia coli* biomass composition [25–27] by using previously published and experimental data from *C. salexigens* [7, 24].

Appropriate amino acid molar ratio for both biomass reactions were calculated assuming that all proteins encoded by *C. salexigens* genome were expressed equally [26, 28]. The proportion of total proteins in response to salinity was calculated based on previous results by our group [7] and the molar ratio of membrane phospholipids in response to salinity were previously determined by Vargas and co-workers [24]. The percentage of G+C in *C. salexigens* genome was experimentally estimated [1]. This was utilized to calculate the genome nucleotide composition, which was used to establish the deoxyribonucleotide molar ratio. The ribonucleotide composition was deduced from the composition of *C. salexigens* rRNA and tRNA in the proportions defined previously [26]. Generally, the relative composition of the nucleic acids does not change with osmolarity, as the overall DNA and RNA content related to dry weight does not seem to be affected by osmotic stress [27]. This assertion was assumed when establishing the relative composition of DNA and RNA. The proportions of the other compounds were assumed not to vary between *E. coli* and *C. salexigens*, and these coefficients were derived from values reported in the literature [25]. Ectoine and hydroxyectoine were included in the biomass composition, because they are growth-associated metabolites and are accumulated in the cells in response to salinity [3]. The coefficients for these compounds were determined based on previous results [7]. Units of each component of a biomass reaction were mmol gDW^−1^ (milimoles per gram cell dry weight). Flux through a biomass reaction has h^−1^ units and is equivalent to the exponential growth rate of the organism [23]. Detailed information on the biomass composition calculation is included in Additional file 1: Tables S1 and S2.

### Conversion of the reconstruction into a mathematical model

The reactions and their participating metabolites in the metabolic network were connected via the stoichiometric matrix (S), which contains the stoichiometric coefficients for each metabolite (row) in each of the reactions of the metabolic model (column). The values in this matrix correspond to the stoichiometry of the metabolite in the reaction. A negative number represents consumption and a positive number represents production of the metabolite. This matrix is the base of the metabolic model that considers metabolite mass conservation and that the accumulation of metabolites (*b*) in the system is equivalent to the product of S matrix and the vector of reaction fluxes (*v*): *S·v* = *b*. When using FBA to solve the differential equations system, one of the requirement is that the organism is in steady-state growth so, *b* = 0, resulting in a linear system of equations [10]. Then the system is bound to a large solution space which needs to be constrained by applying upper and lower bounds to each individual reaction flux (v_i_): *v*_*i* lower_ ≤ *v*_*i*_ ≤ *v*_*i upper*_. Thus, in this process, system boundaries were defined, converting the general reconstruction of *C. salexigens* into a specific model. The initial model differed in scope and boundaries from the final model, which was accomplished after multiple iterations of validation and refinement, and which was used to simulate phenotypic behaviour in a prospective manner. The upper and lower bounds were defined for each reaction based on the flux they could carry. Reversible reactions have an upper bound value of 1000 mmol gDW^−1^ h^−1^ and a lower bound value of − 1000 mmol gDW^−1^ h^−1^, making them practically unconstrained, while irreversible reaction have a lower bound value of zero. By default, the biomass reaction was set as the objective to be maximized. All exchange reactions have zero lower bound value except for the carbon source (glucose, − 10 mmol gDW^−1^ h^−1^), and oxygen (− 20 mmol gDW^−1^ h^−1^), as simulations were run considering growth on a glucose mineral medium in aerobic conditions. Upper and lower bounds of exchange reactions for inorganic ions required by biomass reaction were settled as 1000 and − 1000 mmol gDW^−1^ h^−1^, respectively. The default value for lower bound of glucose uptake was based on the typical glucose uptake rates [25]. Biomass reaction was set as objective function and was maximized to calculate the feasible flux distribution. The obtained value for this reaction was equal to maximum cell growth rate [29].

The final reconstruction metabolic network was named *i*FP764 according to the current standard nomenclature [30]. The complete *i*FP764 model is included in the Additional file 2.

All model simulations were performed using COBRA Toolbox 1.8 [31] in MATLAB (R2014b) (The MathWorks Inc., Natick, MA), linked to the GLPK or Gurobi linear program solver.

### Computational network analysis

#### Minimal medium simulation

The minimal medium M63 [32] was simulated in silico by allowing entrance of some simple salts and ions, water, and O_2_ (aerobic simulations) in the system. For that purpose, upper and lower bounds of exchange reactions for those metabolites exchange reactions were settled as 1000 and − 1000 mmol gDW^−1^ h^−1^, respectively. The composition of the M63 medium is described in Additional file 1: Table S3. The carbon and nitrogen source was changed according to the objective of simulation as well as the secretion rate of pyruvate and acetate (Additional file 1: Table S4).

#### Gap analysis

All blocked metabolites in the *i*PF764 model were identified by using the GapFind MILP algorithm [33] from the COBRA Toolbox. GapFind was run twice, once with each mass balance constraint option. Root no-production and no-consumption metabolites were identified from the model stoichiometric matrix (S) by searching for rows containing only negative or positive coefficients, respectively. Downstream no-production and upstream no-consumption gaps were identified by removing the root gaps from the GapFind outputs [34].

#### In silico validation of GEM model: prediction of C. salexigens growth phenotype

To validate the model for the prediction of growth phenotypes of *C. salexigens,* the ability to utilize different compounds as the sole carbon sources for growth was determined. For this purpose, the lower bound of glucose exchange reaction was constrained to zero, the lower bound of each different carbon exchange reaction, one at a time, was set to − 10 mmol gDW^−1^ h^−1^ (a typical uptake rate for growth-supporting substrates), and growth was maximized by FBA. The target substrate was considered growth supporting if the predicted growth rate was above zero. The simulations were carried out in minimal medium using BIO_L as biomass equation (see Additional file 1: Tables S2, S4). The results arising from the simulations were compared with experimental data [1] and used to validate the model.

#### Gene essentiality

To assess the robustness of the metabolic network to genetic perturbations (e.g., knock-out mutations), we carried out an in silico analysis of the essentiality of single genes and reactions. This enabled us to identify the most fragile nodes of the *i*FP764 network. Gene essentiality simulations were performed by systematically removing each gene from the network and in silico assessing (via FBA) the ability of the model to produce biomass in minimal medium with a sole carbon source (at low and high salinity). In terms of the metabolic simulations implemented in this study, no-growth following perturbation (deletion of gene-associated reaction) indicates the complete disruption of robustness in *C. salexigens* and indicates that this gene/reaction is essential for growth.

#### Simulation at low and high salinity conditions

The effect of salinity over *C. salexigens* metabolism at low and high salinity (conditions for low and high ectoines production, respectively) was examined by flux balance analysis (FBA). Rates for uptake and secretion of metabolites in minimal medium M63 with 0.6 M or 2.5 M NaCl previously described [7], were used as constraints to simulate aerobic growth at both conditions. For this purpose, metabolites present in the medium were first allowed to enter and leave the network by setting the flux limits on the corresponding exchange reactions _vi, min_ > − 1000 mmol gDW^−1^ h^−1^ and _vi, max_ < 1000 mmol gDW^−1^ h^−1^. Second, the glucose and ammonium uptake rates, and the acetate and pyruvate secretion rates, were changed according to experimentally measured fluxes at those salinities, and a biomass reaction (“BIO_L” or “BIO_H”) was selected depending on the salinity simulation conditions.

#### Calculation of fluxes distribution at high and low salinity by Monte Carlo Random sampling

The steady-state flux space is a finite polytope containing all possible steady-state flux distributions [35]. Thus, computing the exact volume for large, *n*-dimensional polytopes is a hard problem [34]. However, it is possible to solve systems of low dimension to examine the accuracy by Monte Carlo approximations. The exact polytope volume calculation algorithm [36] was implemented in Matlab (Mathworks Inc, Massachusetts).

The first step in the Monte Carlo calculation was to determine all the independent variables in the system. This essentially ‘projects’ the flux polytope into a full-dimensional object. The full-dimensional polytope was sampled uniformly over the independent variables, and the dependent variables were back-calculated based on the dependencies in the stoichiometric matrix, S. The result was a random flux distribution, V. The next step was to determine if the flux distribution was valid. Back-calculating the dependent flux values often results in invalid flux distributions (i.e. negative flux values or flux values that violate the *Vmax* constraints). A valid flux distribution was included in the sampled set. Once a set of approximately 20,000 valid points were collected, the process of determining the fractional volumes could start. These valid points were all part of the ‘base’ flux polytope. In this work, Monte Carlo Random Sampling was carried out using the BIO_L and BIO_H to verify the influence of the composition of the formula of the biomass on the internal flows. In addition, specific- salinity constraints based on previous experimental data [7] (Additional file 1: Table S4) were imposed, and the fraction of points meeting the new criteria determined the reduction in volume. The set of all possible flux values for each reaction were represented as a distribution histogram. Each histogram showed one-dimensional information on its *x* axis, in terms of the extent of possible values for that particular flux. The *y* axis represents the “size” of space in the other *r*–1 dimensions resulting from slicing the metabolic solution space along a specific value of the flux through the indicated reaction [35]. The median of the values obtained was normalized by the salinity-specific glucose uptake rate, and was used to analyze the internal flows in all scenarios calculated.

The variation in the flux distribution between both conditions (low and high salinity) was evaluated. For that purpose, flux vector values obtained in the sampling for each reaction in both conditions were compared and evaluated by a Wilcoxon rank sum test. Those reactions which showed significant different distribution (Z statistic over 50 and p value < 0.01) were selected. Additionally, to see the extent of those differences, 2000 points from each reaction vector were randomly selected from the distribution values obtained after sampling in both conditions. Those random points were subtracted to each other between conditions. Then, the average from all computed differences for each reaction was considered as an estimate of flux distribution variation between both conditions.

#### Correlation analysis of metabolic fluxes at high and low salinity

To detect correlated reaction sets in the metabolic network within each condition, a Spearman Rank correlation matrix was performed taking as an input the 20,000 values of each reaction flux vector obtained during the Monte Carlo sampling. A Spearman Rank correlation value over 0.7 or below − 0.7 was considered as the minimal threshold to select reactions that were correlated to each other, respectively. The resulting filtered adjacency matrix was visualized and analyzed in CYTOSCAPE 3.2. as a correlation network, were each node represented a metabolic reaction and each edge represented an observed correlation. Nodes were also grouped according to their membership to different pathways subsystems, which were defined in the metabolic reconstruction *i*FP764.

## Results and discussion

### Construction of the high-quality metabolic model *i*FP764

**Fig. 1:**
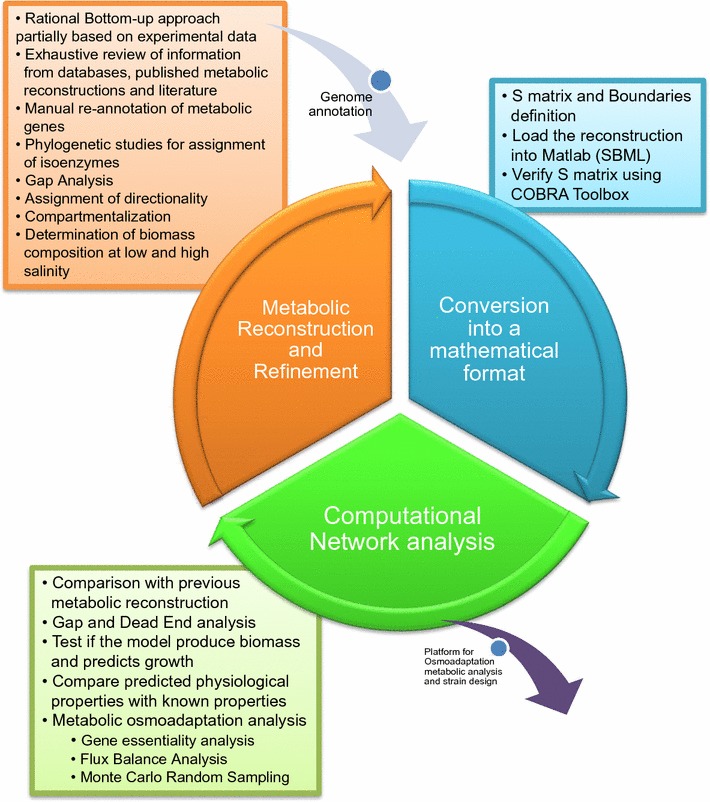
Scheme of the *C. salexigens* metabolic network reconstruction and analysis process

The iterative process to build up the metabolic reconstruction of the *C. salexigens* was based on a bottom-up approach and exhaustive manual refinement of the reconstruction that systematizes the current knowledge about the metabolism *of C. salexigens.* A major value of a manual model-building effort is the careful compilation of genes and reactions by revision of the literature and experimental data, BLAST searches, phylogenetic analysis, database comparison, gaps filling, etc., to achieve a robust metabolic model with a highly reliable prediction capacity. All the workflow for the *C. salexigens* metabolic reconstruction followed standard procedures described in the literature [23], and is detailed in "Methods" and Fig. 1. The final reconstruction metabolic network was named *i*FP764 according to the current standard nomenclature [30]. The most relevant steps are described in detail in (Additional file 1).

#### Compartmentalization

In *C. salexigens*, the differential use of the periplasmic or cytoplasmic variants of the Entner–Doudoroff pathway contributes to modulating the production of reducing equivalents (NADPH), adapting it to the requirements for ectoine synthesis [7]. For this reason, the *C. salexigens* metabolic model *i*FP764 generated in this work included three distinct cellular compartments: the cytoplasm, the periplasm and the extracellular space.

**Fig. 2:**
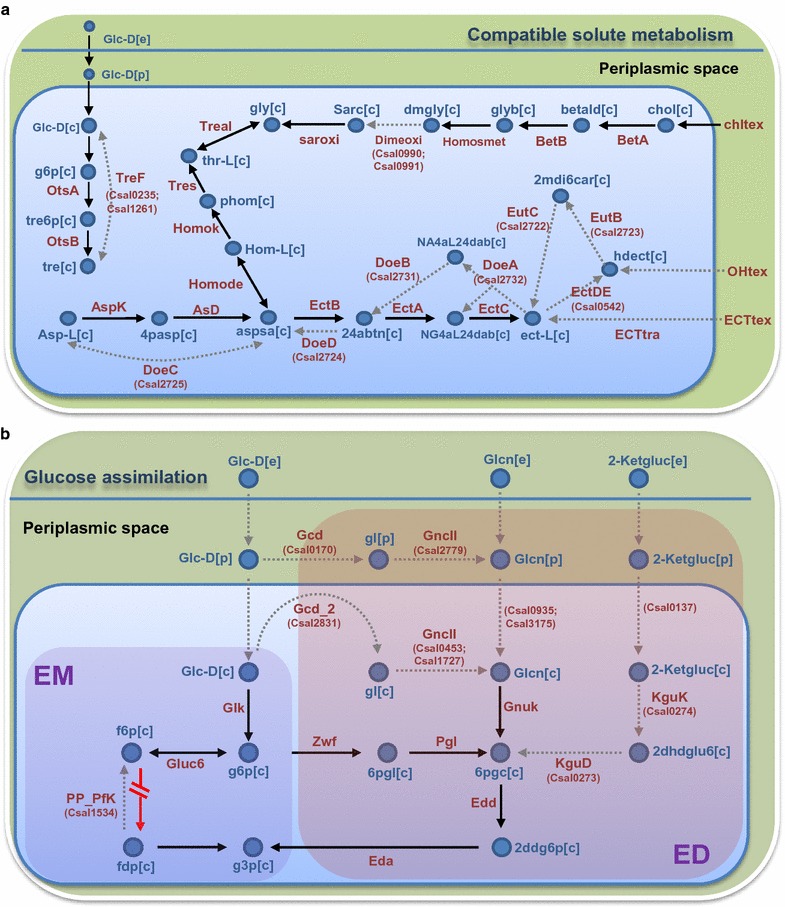
Examples of relevant curated pathways in *C. salexigens*. **a** Pathways related with compatible solutes metabolism. **b** Entner–Doudoroff (ED) and partial glycolysis (EM) pathways of central metabolism. Names of reactions and associated genes are shown in red. A discontinuous arrow means a new reaction or a re-annotated gen-associated reaction in *i*FP764, with respect to *i*OA548. A red-truncated arrow represents a gene-associated reaction non-included in *i*FP764, respect to *i*OA548. **a** glc-d[c]: d-glucose; f6p[c]: beta-d-fructose-6-phosphate; tre6p[c]: alpha, alpha-trehalose 6-phosphate; tre[c]: alpha, alpha, trehalose; chol[c]: Choline; dmgly[c]: *N,N*-dimethylglycine; sarc[c]: Sarcosine; gly[c]: Glycine; thr-l[c]: l-Threonine; phom[c]: *O*-phospho-l-homoserine; hom-l[c]: l-homoserine; asp-L[c]: l-aspartate; aspsa[c]: l-aspartate-4-semialdehyde; 4pasp[c]: 4-phospho-l-aspartate; 24abtn[c]: l-2-4-diamino-butanoate; ect-l[c]: ectoine; 2mdi6car[c]: 2-ethyl-4,5-dihydropyrimidine-6-carboxylate; hdect[c]: 5-Hydroxyectoine; nA4aL24dab[c]: *N*-alpha-acetyl-l-2,4-diaminobutyrate; nG4aL24dab[c]: *N*-4-Acetyl-l-2,4-diaminobutanoate. **b** glc-d[c]: d-glucose; f6p[c]: beta-d-fructose-6-phosphate; fdp[c]: beta-d-fructose-1,6-bisphosphate; g6p[c]: d-glucose 6-phosphate; g3p[c]: glyceraldehyde-3-phosphate; gl[c]: d-glucono-1,5-lactone; 6pgl[c]: d-glucono-1,5-lactone 6-phosphate; glcn[c]: d-gluconic-acid; 6pgc[c]: 6-phospho-d-gluconate; 2ddg6p[c]: 2-Dehydro-3-deoxy-6-phospho-d-gluconate; 2-ketgluc[c]: 2-keto-d-gluconic acid; 2dhdglu6[c]: 6-phospho-2-dehydro-d-gluconate

This compartmentalization allowed the inclusion of transport systems in both, the inner and outer membrane, and represents more accurately the metabolic machinery available in *C. salexigens,* including pathways for compatible solute metabolism, and associated central metabolism (Fig. 2a, b). Apart from periplasmic oxidation of glucose to gluconate, the most important routes included in the model were those involved in the transport of d-glucono-1,5-lactone (gl), and 2-keto-d-gluconate (2ddglcn), two compounds that can be used by *C. salexigens* as sole carbon sources [7] (Table 2, Fig. 2b).

**Table 2:** Properties of metabolic reconstructions of *C. salexigens*

Features		
In silico metabolic model	*i*FP764	*i*OA584 [42]
No. of total reactions	1530	1387
No. of total ORFs associated	1190	874
No. ORFs no associated	340	513
No. of total metabolic reactions	1023	880
Multiple compartments*	33	0
Cytoplasmatic	943	880
Periplasmic	42	0
Extracellular	5	0
No. of total transport reactions	507	507
Exchange	178	507
Extracellular to periplasmic	149	0
Periplasmic to cytoplasmic	137	0
Periplasmic to extracellular	6	0
Cytoplasmic to periplasmic	34	0
Extracellular to cytoplasmic	3	0
No. of total metabolites	1123	1411
Cytoplasmatic	790	920
Periplasmic	176	0
Extracellular	157	491
Unique functional proteins	764	579
Single gene	458	579
Genes involved in complexes	155	0
Instances of isozymes	151	0

* Reactions can occur in or between multiple compartments and metabolites can be present in more than one compartment

#### Formulation of two biomass reactions to simulate low salinity and high salinity conditions

Two specific biomass reactions, “BIO_H” and “BIO_L”, were formulated to simulate high and low salinity conditions, respectively, and incorporated into the reconstruction. The formulation of both reactions was partially based on previous *C. salexigens* experimental data, and on the formulation defined for *Escherichia coli* [28]. Ectoine and hydroxyectoine were included in both biomass reactions because they are growth-associated metabolites [3], and their specific contents are affected by the salinity of growth medium [7]. This provided two condition-specific models that will serve as platforms to test metabolic capabilities at low and high salinity. Detailed information on the biomass composition formulation can be found in “Methods” and Additional file 1: Tables S1 and S2.

#### Properties of the iFP764 reconstruction

**Fig. 3:**
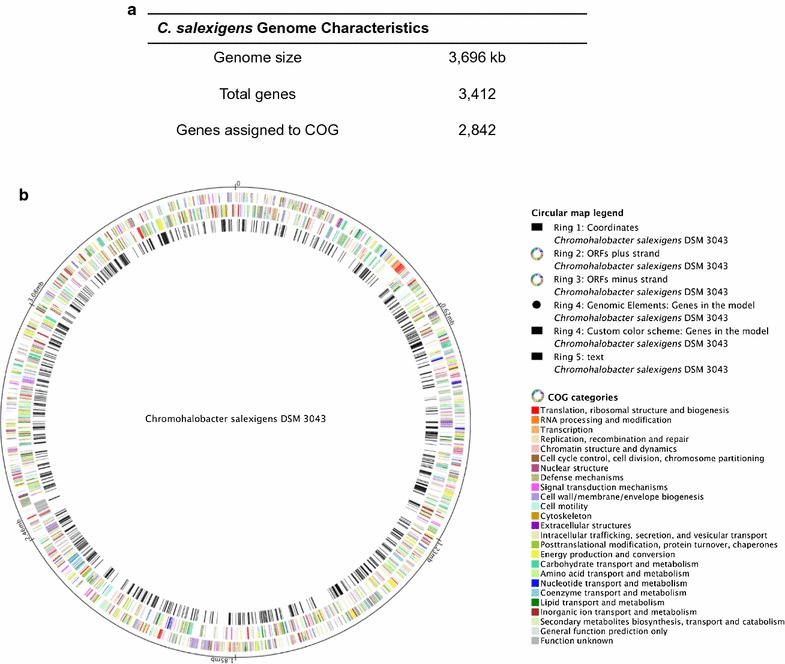
**a** Statistic of *C. salexigens* genome. **b** Genomic coverage and COG assignment of the metabolic genes included in *i*FP764

The genome of *C. salexigens* is composed of a circular chromosome of 3696 kb encoding 3412 total genes [6] (Fig. 3a). The *i*FP764 reconstruction accounts for 764 genes, 1530 reactions and 1123 metabolites segregated into 3 compartments: cytoplasmic, periplasmic and extracellular. The metabolic reactions include 943 cytoplasmic reactions, 42 periplasmic reactions, 5 extracellular reactions and 33 multiple compartments reactions (Table 2). The *C. salexigens* reconstructed metabolic network presented in this work covers 22.30% of the total genes present in the genome (Fig. 3b). The coverage of the genome is similar to those of metabolic networks reconstructions published, such as those of *Pseudomonas aeruginosa* (19.3% coverage; [37]), *Escherichia coli* (32% coverage; [27]), *Bacillus subtilis* (20% coverage; [38]), *Clostridium acetobutylicum* (13.0% coverage; [39]) and the halophilic bacterium *Halomonas smyrnensis* AAD6T (25.3% coverage; [40]). All enzymes included in the *i*FP764 model were clustered into one of the 12 Orthologous Groups (COGs) functional categories of proteins [41] (Fig. 3b).

### Comparison of *i*FP764 with the previous *C. salexigens* metabolic reconstruction *i*OA584

After completion of the *i*FP764 metabolic network reconstruction, it was compared with the previous semi-automatically-generated, reconstruction of *C. salexigens* (*i*OA584) [42]. Table 2 details the number of reactions of *i*FP764 in each subsystem and the comparison with the previous reconstruction *i*OA584. Up to 169 genes that were found in the *i*OA584 model were not include in the *i*FP764 model, since these routes have not been described in *C. salexigens,* and the reactions catalyzed by these enzymes were not connected to any pathway.

Whereas periplasmic and extracellular metabolic reactions were considered in *i*FP764, *i*OA584 did not include any transport reaction other than exchange reactions. In addition, the semi-automated reconstruction contains just single genes associated with reactions, but genes associated to enzymatic complexes or isoenzymes were not annotated, as in *i*FP764. This improvement is very relevant to implement strategies for in silico knockout analysis or strain design for metabolic engineering.

**Fig. 4:**
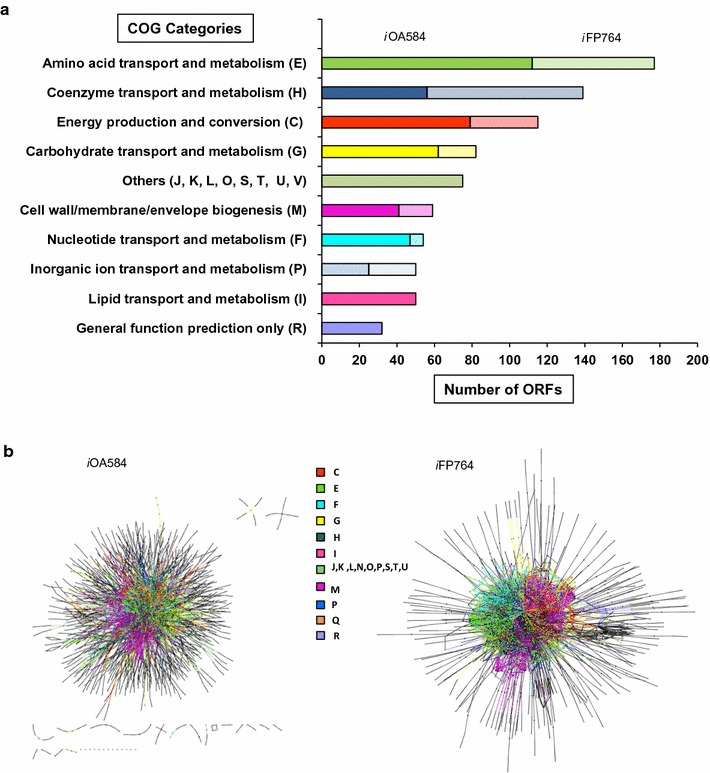
Comparison of *i*FP764 and *i*O548 metabolic networks of *C. salexigens*. **a** Distribution of genes in each of 10 functional categories (COG) in the *i*OA584 and *i*FP764 metabolic networks. Each functional category was represented with one color. The number of new genes associated to reaction (ORF) in the *i*FP764 are indicated by the lighter portion at the far right side of each bar. **b** Graphic representation of *i*OA584 (left) and *i*FP764 (right) metabolic networks. The colors and letters represent the functional categories of genes (COG) associated to reactions. Nodes are gene-enzyme associated reactions (squares) and metabolites (circles). Edges are represented by arrows which indicate the participation of metabolite in a reaction and its directionality (consumed or produced). The representation was performed by using Cytoscape (3.2). The connectivity problem in the *i*OA584 model is evidenced by the disconnected reactions and metabolites from the central network

A detailed comparison of the number of ORFs assigned from each COG functional category in both models is presented in Fig. 4a. The largest increase in coverage compared with the previous reconstruction corresponded to transport and metabolism of amino acids (61% increase, with addition of 67 ORFS). Additionally, complete synthesis and degradation pathways of the main compatible solutes of *C. salexigens*, which are necessary for osmoadaptation and drive metabolic adaptation to salinity, were included in *i*FP764 (see Additional files 1, 2), whereas some of them were found wrong-gene associated or not present in *i*OA584.

#### Gap analysis

The final reconstruction *i*FP764 included some incomplete metabolic pathways that contained gaps or blocked reactions, which generated 98 dead-end metabolites: 27 root no-production metabolites (metabolites that participates in consuming reactions but not in producing reactions) and 71 no-consumption metabolites (metabolites that are present in producing reactions but not in consuming reactions) (Additional file 1: Table S5). In total, 11.17% of the metabolites in *i*FP764 were blocked under all conditions due to gaps. The major root no-production metabolites were those implicated in the production of those, like tRNAs, serve for non-metabolic functions once they are produced. This problem is also present in the *E. coli i*JO1366 metabolic reconstruction [25]. On the other hand, out of the 71 no-consumption metabolites detected mainly belonged to pathways related to tRNA (9.2%), cell envelopes, including cell wall and cell membranes (15.6%), cofactors and prosthetic groups synthesis (8.6%) or carbon metabolism (7.1%). With the exception of the metabolites found in the routes of carbon metabolism, the rest of dead ends metabolites were found in pathways insufficiently described in *C. salexigens*. An important flaw in the automatically generated *i*OA584 was the high number of gaps and dead ends metabolites, as we found 272 no-production metabolites and 645 no-consumption metabolites.

The effort in the exhaustive refinement process leading to the *i*FP764 metabolic network resulted in an improvement of connectivity compared with *i*OA584. A graphic representation of both networks, performed using CYTOSCAPE, evidenced a connectivity failure in the *i*OA584, as several disconnected reactions and dead-end metabolites from the central network were found. This was highly solved up in *i*FP764 (Fig. 4b).

#### Biomass production

Even the most complete models are not definitive, if they contain gaps or missing information [25]. However, filling in the gaps as much as possible is important to make the model more realistic, improving the predictive capability, and this degree of completion is especially needed when the model does not predict growth under some specific condition (i.e., given sets of nutrients and secretions) when submitted to FBA simulation [43]. This occurs due to the fact that the reactions producing or consuming dead-end metabolites can never carry flux under any condition, so one fundamental requirement for a reconstructed metabolic network is its ability to produce flux through the biomass reaction (i.e., to predict growth) [44]. When the *i*OA584 model was analyzed by FBA with the COBRA Tool box, it could not produce biomass, and consequently it could not simulate cell growth, in contrast to *i*FP764. This is likely due to the numerous flaws present in the automated network that are absent (or, at least, present in a much lower number) in the curated *i*FP764 model. Among these, incorrect reaction stoichiometry, repetitions, dead-end metabolites, problems with proton balances, and severe troubles in connectivity, were found in *i*OA584.

In summary, in addition to the enlargement, this comparison revealed great differences between the two models, and a large number of drawbacks associated to semi-automatically-generated networks.

### Model validation

Flux balance analysis (FBA) can be used with a constraint-based model to predict metabolic flux distributions, growth rates, and substrate uptake and production secretion rates. Thus, FBA analyses of the model with a set objective function (i.e. biomass reaction that is equal to growth) to generate maximal amounts of biomass precursors (i.e., simulated optimal growth) from substrates available in growth media can generate results that are quantitatively consistent with experimental data [25, 33]. In this context, the utilization of different carbon sources by the *i*FP764 network was simulated and compared to previous reports [1, 7].

The growth in minimal medium M63 was simulated by fixing the set values for the exchange reactions for the import of metabolites present in the simulated growth medium. Each substrate was taken as the sole carbon and energy source in the simulation and the uptake rates were all set to the same value (10 mmol gDW^−1^ h^−1^) in aerobic conditions. The biomass reaction utilized for the validation was BIO_L.

For 35 experimentally tested carbon sources, 85% agreement was found between the predicted and experimentally tested utilization of substrates by *C. salexigens* [1]. This included the inability to use l-methionine and l-valine (Table 3). The qualitative evaluation of the predictive capability of the model reconstructed herein falls within the range of most metabolic reconstructions available to date [37, 45].

**Table 3:** In silico prediction of utilization of various metabolites as carbon sources and comparison with experimental data

Metabolite name	Compound	Metabolite formula	In silico simulation	Experimental data	References
2-ketogluconate	2ddglcn[e]	C_6_H_9_O_6_	+	+	Arahal et al. [1]
Acetate	ac[e]	C_6_H_9_O_7_	+	+	Arahal et al. [1]
d-Alanine	ala-d[e]	C_3_H_7_NO_2_	+	+	Arahal et al. [1]
l-Arginine	arg-l[e]	C_6_H_15_N_4_O_2_	+	+	Arahal et al. [1]
l-Asparagine	asn-l[e]	C_4_H_8_N_2_O_3_	+	+	Arahal et al. [1]
Choline	chol[e]	C_5_H_14_NO	−	+	Arahal et al. [1]
Citrate	cit[e]	C_6_H_5_O_7_	+	+	Arahal et al. [1]
Ectoine	ect-l[e]	C_6_H_10_N_2_O_2_	+	+	Vargas et al. [24]
Ethanol	etoh[e]	C_2_H_6_O	+	+	Arahal et al. [1]
Fructose 6-phosphate	f6p[e]	C_6_H_11_O_9_P	+	+	Data not shown
d-Fructose	fru[e]	C_6_H_12_O_6_	+	+	Arahal et al. [1]
Fumarate	fum[e]	C_4_H_2_O_4_	+	+	Arahal et al. [1]
d-Glucose	glc-d[e]	C_6_H_12_O_6_	+	+	Arahal et al. [1]
d-Gluconate	glcn[e]	C_6_H_11_O_7_	+	+	Pastor et al. [7]
l-Glutamine	gln-l[e]	C_5_H_10_N_2_O_3_	+	+	Arahal et al. [1]
l-Glutamate	glu-l[e]	C_5_H_8_NO_4_	+	+	Arahal et al. [1]
Glycine	gly[e]	C_2_H_5_NO_2_	+	+	Arahal et al. [1]
Glycine betaine	glyb[e]	C_5_H_11_NO_2_	−	+	Arahal et al. [1]
Glycerol	glyc[e]	C_3_H_8_O_3_	+	+	Arahal et al. [1]
5-hydroxyectoine	hdect[e]	C_6_H_10_N_2_O_3_	+	+	Vargas et al. [24]
d-Lactate	lac-d[e]	C3H5O3	+	−	Arahal et al. [1]
l-Lactate	lac-l[e]	C_3_H_5_O_3_	+	−	Arahal et al. [1]
Lysine	lys-l[e]	C_6_H_15_N_2_O_2_	−	+	Arahal et al. [1]
d-Malate	mal-d[e]	C_4_H_4_O_5_	+	+	Arahal et al. [1]
l-Malate	mal-l[e]	C_4_H_4_O_5_	+	+	Arahal et al. [1]
Maltose	malt[e]	C_12_H_22_O_11_	+	+	Arahal et al. [1]
d-Mannose	man[e]	C_6_H_12_O_6_	+	+	Arahal et al. [1]
l-Methionine	met-l[e]	C_5_H_11_NO_2_S	−	−	Arahal et al. [1]
Proprionate	ppa[e]	C_3_H_5_O_2_	+	+	Arahal et al. [1]
l-Proline	pro-l[e]	C_5_H_9_NO_2_	+	+	Arahal et al. [1]
d-Ribose	rib-d[e]	C_5_H_10_O_5_	+	+	Arahal et al. [1]
l-Serine	ser-l[e]	C_3_H_7_NO_3_	+	+	Arahal et al. [1]
Succinate	succ[e]	C_4_H_4_O_4_	+	+	Arahal et al. [1]
Trehalose	tre[e]	C_12_H_22_O_11_	+	+	Arahal et al. [1]
l-Valine	val-l[e]	C_5_H_11_NO_2_	−	−	Arahal et al. [1]

(+) Metabolized (−) not metabolized

Disagreement between in silico simulations and literature reports was found for five compounds. On the one hand, choline, lysine and glycine betaine support *C. salexigens* growth, according to experimental data, whereas the simulation did not predict growth with these compounds. Conversely, the *i*FP764 model predicted growth with L-lactate and D-lactate, which is in disagreement with experimental data [1], as *C. salexigens* is not able to grow with any lactate isomer. Disagreements between the computational and experimental data are acceptable and can be divided into two main categories. Instances where experimental growth is observed and no growth is predicted computationally (i.e. with choline, lysine, or glycine betaine) points to areas where further biochemical characterization is needed and define targeted areas for biological discovery [28]. In contrast, cases in which computational growth is predicted and not observed experimentally (i.e., with lactate) indicate possible areas where there are either errors in the reconstruction or, alternatively, where regulation limits the utilization of pathways needed for growth.

### Theoretical analysis of *C. salexigens* physiological capabilities

#### Growth in different carbon sources

The interest of the in silico prediction is to extend the knowledge about which pathways are active, and for which processes and to what extent different substrates are utilized by the organism. This information can be used to guide labeling studies as well as to interpret results from experiments using similar conditions such as gene and protein expression data. The *i*FP764 computational model contains exchange reactions for 90 different compounds. It is therefore possible to use *i*FP764 to predict the growth capabilities of *C. salexigens* on a very wide range of media conditions. As a demonstration, FBA was used to forecast growth on the 90 possible carbon sources under aerobic conditions using *i*FP764. As shown in Additional file 1: Table S6, the *i*FP764 model predicted that 51 out of these 90 carbon sources are growth supporting.

#### Essentiality of genes and reactions depending on salinity

One of the major applications of GEMs is improving the performance of microbial cell factories by a system wide analysis of the metabolic network. Robustness is generally defined as the cellular ability to maintain biological processes against internal and external perturbations, including disruption of genes and inactivation of enzymes (e.g., knock-out mutations) [46].

In this regard, *i*FP764 robustness is crucial for predicting ectoine production under certain conditions. Thus, reaction essentiality simulations were performed by systematically removing each gene from the network and assessing the ability of the model to predict growth in minimal medium with glucose as carbon source. These FBA simulations were conducted at low and high salinity conditions (See "Methods").

**Fig. 5:**
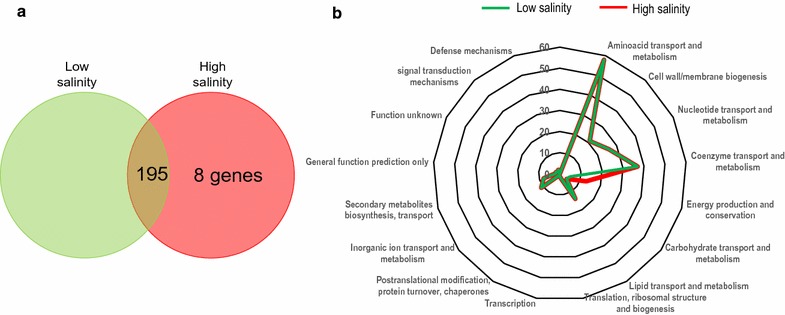
Influence of salinity in gene essentiality of *C. salexigens*. **a** Comparison of the number of essential gene at low and high salinity. **b** COG distribution of essential genes at low and high salinity

The study of gene essentiality revealed that 26.6% and 25.5% of the total 764 genes included in *i*FP764 were essential for cell growth at high and low salinity, respectively (see Additional file 3: Table S7 for a complete list). Most of these genes (around 97%) were also included in the CGE database of essential gene clusters [47], providing reliability to our prediction. A total of 195 genes were essential in both conditions, but 8 genes (*csal_3283* to *csal_3290*) were essential only at high salinity (Fig. 5a), corresponding to the ATP synthase complex subunits. This finding underlines a relevant role of bioenergetics in the metabolic adaptation to salinity. It may be due to the fact that life at high salt concentration is energetically expensive, since it involves the build up and maintenance of steep ion concentration gradients across the cell membrane and compatible solute synthesis, which is a very energetically and metabolically demanding process [48].

### Analysis of metabolic osmoadaptation by FBA

In the previous section, we capitalized on the current knowledge of *C. salexigens* metabolism to compare the in silico prediction of the model with physiological data. Here, we used FBA to investigate the metabolic adaptation of *C. salexigens* to high and low salinity by using our metabolic network (*i*FP764).

**Fig. 6:**
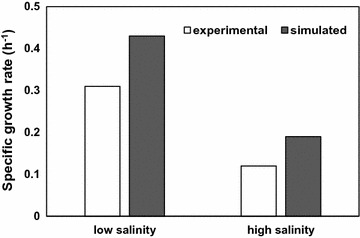
Comparison of the in silico and experimentally determined growth rate at low and high salinity

Recently, Pastor and co-workers [7] described the central metabolism of *C. salexigens* at different salt concentrations. They showed that salinity influences glucose consumption, pyruvate and acetate excretion and biomass production. They also showed that pyruvate and acetate excretion was the result of an overflow metabolism derived mainly from the metabolic “rigidity” of a certain number of reactions (all of them related to providing TCA cycle metabolites for the synthesis of ectoines). It was proposed that this rigidity (i.e., the absence of variation in the metabolic ratios of these enzymes), led to this overflow at low salinity. The in silico growth rate on glucose minimal medium at high and low salinity was calculated by FBA (see simulation parameters in Additional file 1: Table S4) and compared with the experimental growth rates [7] (Fig. 6). If the in silico rate is lower than the experimental, it would indicate that the network may lack important reactions that influence the efficiency of conversion of the carbon source into biomass constituents and/or energy [49]. However, the calculated in silico rate (0.43 and 0.19 h^−1^) were higher than the experimental rates observed by [7] (0.31 and 0.12 h^−1^ at low and high salinity, respectively) (Fig. 6). This greater efficiency of the in silico model versus in vivo growth data is also consistent with studies that suggest that the optimal growth objective function does not necessarily reflect the real functioning of biochemical networks over a wide range of environmental conditions [49–51]. Moreover, this finding suggests that some of the processes included in the network might be unrealistically efficient and/or that *C. salexigens* may be diverting resources into other processes not accounted for in the model, or that others additional constrains might be taken into account when performing simulations. Thus, although acetate and pyruvate and gluconate were identified by HPLC–MS as the major by-products of glucose metabolism [7], it may be possible that other non-measured products different of acids and alcohols could be secreted by *C. salexigens*. Additionally, we have in silico observed a highest consumption of O_2_ (relative to glucose consumption) (− 2.66) and a greater production of CO_2_ (2.6) at high salinity when compared with low salinity (1.4 and 1.35, respectively). This fact reflects differences in oxidative metabolism between high and low salinity in *C. salexigens.* This could be related with differences in rates of TCA cycle activity [7] and, consequently, it might be reflected in variations in O_2_ consumption and CO_2_ production. So, it would be very interesting to deepen into this question experimentally in the future, and use these data as additional constraints to accurate growth rate predictions. In this way, the use of the *i*FP764 model would be a very useful tool to drive additional experiments.

The in silico growth rate is influenced not only by the structure of the metabolic network, but also by other factors including biomass composition and the use of constraints. Therefore, since both the biomass composition and constraints values vary with salinity, we evaluated the influence of these factors on the predicted growth yield. First, we analyzed the effects of changes in the ratios of biomass components on the *i*FP764 growth yield. This was done by varying only BIO_L or BIO_H when simulating at low and high salinity with no other constraints, and performing FBA with the biomass reaction set as the objective function. These analyses indicated that maintaining the glucose entry at 10 mmol gDW^−1^ h^−1^, without using any other constraints, did not affect growth rate (1.83 and 1.84 h^−1^ at low and high salinity, respectively). Similar results were obtained when simulating with a *Pseudomonas putida* model, a 20% increase of any single biomass constituent had an effect of less than 1% on the growth yield [49]. However, although the growth rate was not affected, the internal fluxes distribution could vary according to the formula of the biomass utilized in each condition. This finding is discussed below.

The second factor influencing the efficiency of the in silico optimal growth is the use of constraints. Metris and co-workers simulated osmotic stress using FBA and different biomass reactions that included compatible solutes as metabolites and their respective salinity-specific proportion [52]. They suggested that this was not sufficient, and that additional constraints were needed to explain the chemical-physical limitations of the growth rate simulation. On the other hand, Covert and Palsson introduced additional constraints using Boolean operators, and this approach was shown to eliminate a large number of extreme pathways [53].

In this work, the simulations performed corroborated previously obtained growth rates in vivo where a higher growth rate was observed at low salinity. This was mainly due to the contribution of the salinity dependent-experimental-based constraints. However, additional experimental constrains could be added in a future to accurate growth rate or fluxes prediction.

### In silico prediction of metabolic flux distributions shifts in response to salinity

The above data show that the *i*FP764 model is coherent and reproduces the major metabolic features of *C. salexigens*. However, FBA can only generate a single flux distribution corresponding to maximal growth under any given environmental condition. An alternative approach is to characterize all flux distributions that are allowed by the mass balance (stoichiometric) and flux capacity constraints. There are a number of methods that allow characterizing entire flux solution spaces, including various forms of pathway analysis [10]. Among them, Monte Carlo sampling method is a rapid and scalable way to characterize the structure of the allowed space of metabolic fluxes. These analyses can provide all possible steady-state distribution for unknown metabolic fluxes and guide making informative experimental measurements [35].

In this work, Monte Carlo sampling was used to calculate the probability of flux distributions for all the reactions of *i*FP764 model in two scenarios. In the first scenario, specific constraints (for glucose and ammonium consumption or pyruvate an overflow metabolites secretion) and biomass composition for high and low salinity, were used to simulate low and high salinity conditions. In the second scenario only specific biomass reactions (but no salinity-specific constraints), were used to simulate both salinities. The set of all possible flux values for each reaction was represented as a flux distribution histogram.

**Fig. 7:**
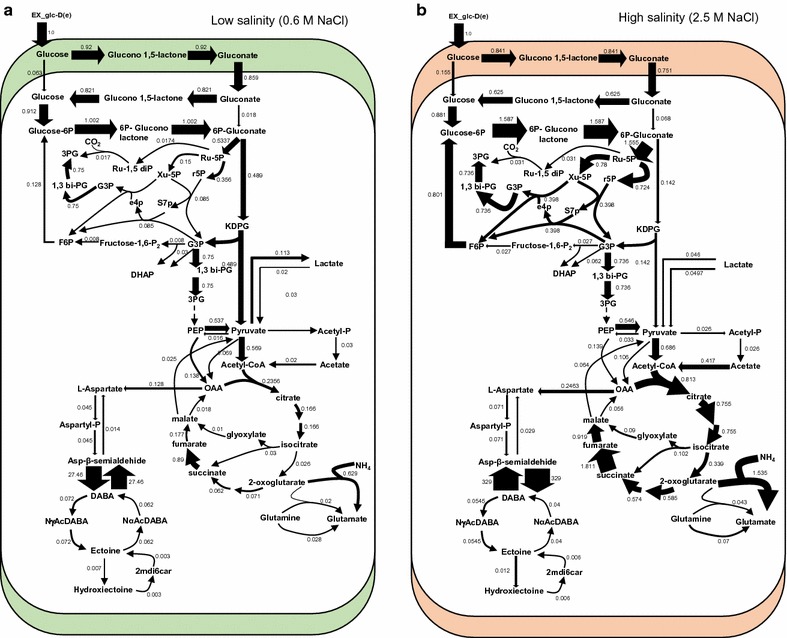
Overview of central and ectoines metabolism fluxes distribution at low and high ectoines production conditions (only the first scenario is shown). Median values of fluxes calculated by Monte Carlo Random sampling for each reaction are normalized relative to the glucose consumption rate of each specific salinity-condition **a** Low salinity (0.6 M NaCl). **b** High salinity (2.5 M NaCl). *Ru-5P* d-ribulose-5-phosphate, *Xu-5P* d-xylulose-5-phosphate, *r5P* d-ribose-5-phosphate, *S7p* sedoheptulose-7-phosphate, *e4p* d-erythrose-4-phosphate, *G3P* glyceraldehyde-3-phosphate, *F6P* fructose-6-phosphate, *1,3 bi-PG* 3-phospho-d-glyceroyl-phosphate, *3PG* 3-phospho-d-glycerate, *PEP* phosphoenolpyruvate, *OAA* oxaloacetate, *DABA* l-2-4-diamino-butanoate; *NαAcDABA N*-alpha-acetyl-l-2,4-diaminobutyrate, *2mdi6car* 2-methyl-4,5-dihydropyrimidine-6-carboxylate

The first scenario revealed different distributions of fluxes at low and high salinity, suggesting the most relevant pathways involved in metabolic osmoadaptation of *C. salexigens* (Fig. 7, Additional file 1: Figure S1). Interestingly, differences in the flux distribution related to salinity of reactions belonging to the pentose phosphate, Entner–Doudoroff, and TCA cycle pathway were observed, supporting our previous hypothesis that salinity affects ATP generation or cell redox balance through a differential reducing power generation, by preferential use of glucose utilization pathways [7]. This analysis also suggests that the existence of alternate pathways for glucose metabolism differing in redox balance may allow the microorganism to control the rate of production of redox equivalents, and to finely tune its redox state to maximize growth and ectoines biosynthesis. This conclusion is supported by the calculation of the theoretical amount of ATP produced per mol of glucose at low and high salinity. According to the *i*FP764 model, the theoretical wild-type ATP yield at high salinity is around 2.6 fold higher compared to low salinity, indicating that energetic metabolism is more efficient at high salinity, as it was experimentally described previously [7]. Indeed the rate of ATP production through oxidative phosphorylation was 3-fold higher at high salinity compared to low salinity, suggesting that this improvement in metabolism efficiency could be driven by an increment in respiration rate.

The second scenario was used to evaluate only the effect of each biomass reactions (BIO_L and BIO_H) on flux distribution. After random sampling, modifications of the flux distribution were also observed, but differences were not as evident as in the first scenario (data not shown). This finding suggests that biomass composition formulated at low and high salinity does not significantly influence the flux distribution under these conditions.

Overall, the above results indicate that our model serves as an excellent platform to analyse metabolic adaptation to salinity. It also indicates that although specific-biomass composition is needed to be accurate when performing simulations, specific-experimental constraints are more relevant and necessary to guarantee that in silico fluxes are close to those experimentally observed.

### Correlation between metabolic fluxes at low and high salinity

Modularization may be a useful concept to understand large metabolic networks by breaking it apart into manageable pieces [54]. The highly correlated clusters may be considered as functional modules, which mainly include groups of enzymes that are jointly needed to obtain a specific product. We analysed the correlation between fluxes of sets of reactions at high and low salinity obtained by Monte Carlo sampling. This analysis provided us significant clues on the correlations between individual reactions and between sets of reactions, probably involved in metabolic adaptation to salinity and/or ectoines demand.

**Fig. 8:**
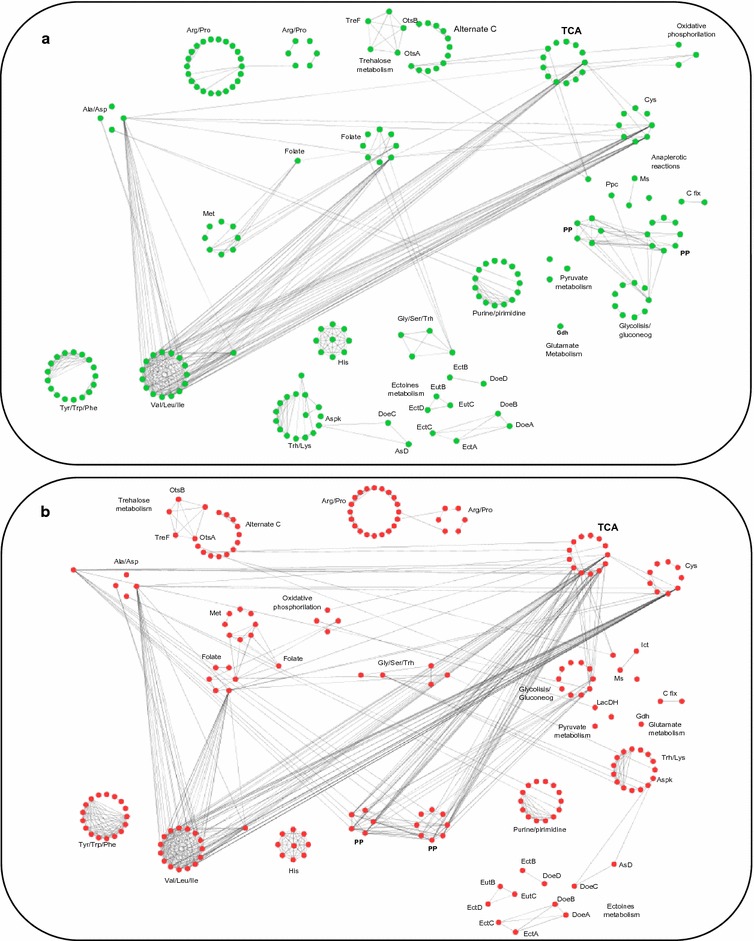
Fluxes correlation network of compatible solute and main carbon and nitrogen metabolism pathways at low (**a**) and high (**b**) salinity. Each node represents a metabolic reaction and each edge represents an observed correlation (with Spearman Rank correlation value larger than 0.7). The adjacency matrix was visualized in CYTOSCAPE (3.2). Nodes were grouped according to their membership to different pathway subsystems and some reactions/enzymes was specified. *Aspk* aspartate kinase, *AsD* aspartate semialdehyde dehydrogenase, *C fix* carbon fixation, *Ict* isocitrate lyase, *Ms* malate synthase, *PP* pentoses phosphate, *Ppc* phosphoenolpyruvate carboxylase

From all interrelations found, we specifically focused our analysis in those clusters related to the metabolism of compatible solutes and associated carbon and nitrogen metabolism (209 reactions for low salinity and 211 for low salinity). In this context, we found a higher number of correlations at high salinity (549) compared to low salinity (461) (Fig. 8).

Regarding nitrogen metabolism, the valine/leucine/isoleucine sub-system was found highly inter-correlated at high and low salinity, both between their internal reactions and also with other subsystems, like the alanine/aspartate, cysteine, TCA cycle, and folate metabolism subsystems. On the contrary, some amino acids subsystems showed to be independent, as they presented a high degree of internal correlation but their reactions did not correlate with other subsystems. This was observed for the histidine and tyrosine/phenylalanine/tryptophan subsystems. This fact occurred in both conditions and no significant variation between the correlation patterns was observed (Fig. 8).

The TCA cycle cluster was more correlated with other clusters at high salinity than at low salinity (Fig. 8a, b). At low salinity, this subsystem showed correlated reactions with the valine/leucine/isoleucine, cysteine, alternate Carbon, and oxidative phosphorylation subsystems. However, at high salinity an important number of additional correlations appeared with other subsystems, like the pentose phosphate (this subsystem included also reactions from ED pathway) and glucolysis/gluconeogenesis clusters. All these clusters were clearly intercorrelated at high salinity (Fig. 8b). Interestingly, the recently described ED-EMP cycle, which recruits activities from the ED, EMP and PP pathways to ensure an appropriate supply of NADPH reducing power for coping with environmental stress [55], is present in *C. salexigens.* Thus, the PPi dependent 6-phosphofructokinase (Csal_1534), acting in the reverse reaction as a bisphosphatase, would supply the lack of Pfk, catalyzing the conversion of fructose 1,6- bisphosphate into fructose-6-phosphate [7], and completing the cycle. However, correlation analysis grouped the ED-EMP cycle reactions in three modules in both salinities. The first cluster was formed by Pgi (GLUC6), Zwf, and Pgl (PGL6), the second cluster was formed by Fda (FRUB) and Fbp, and the third cluster was constituted by the Edd and Eda reactions. Interestingly, the first cluster of reactions [two of them from pentose phosphate (PP) pathway and one of the from EMP pathway], were correlated with some TCA cycle reactions at high salinity [Aconitase (ACOHY2), aconitate hydratase (ACOHY1), Fumarate hydratase (FUM1), and Cs (CITSY)], and with additional reactions of the pentose phosphate pathway. The third cluster of reactions (both of them belonging to the ED pathway, but included in the pentose phosphate subsystem) showed correlation at high salinity with Pdh (Glycolysis/Gluconeogenesis subsystem). The ED-EMP cycle has been suggested to be an essential distributor of carbon through the central metabolic metabolism and an enhanced supply of reducing equivalents [55]. Therefore, the above results could pave the way for future experimental design, in order to figure out how this cycle is functioning in *C. salexigens,* and if it is partially coupled with the TCA cycle at high salinity, where high ectoines demand requires high levels of reducing power and carbon.

Regarding ectoines metabolism, the correlation analysis distributed this subgroup into four cyclic modules. The first module included reactions for the synthesis and degradation of hydroxyectoine (EutC, EutB and EctDE), the second module corresponded to the futile cycle for ectoine synthesis and degradation (EctC, EctA, DoeA, DoeB and EctA) [56], the third module was responsible for the l-aspartate semialdehyde conversion into diamino butyric acid, or viceversa, with an interconversion coupled reaction of glutamate to 2-oxoglutarate (EctB and DoeD). The last module included reactions responsible of the synthesis of L-aspartate to L-aspartate semialdehyde, or viceversa, [DoeC, AsD, aspartate kinase (ASPK)]. Interestingly, all the four modules were independent both at low and high salinity conditions, as they did not correlate to each other, and they were not correlated with other subsystems (Fig. 8).

Regarding other compatible solutes, trehalose synthesis and degradation reactions (OtsA, OtsB and TreF) formed a separate module including internal reactions of central C subsystem, particularly those involved in the conversion of glucose-1-P into UDP-glucose (UTPGLPHO, UTP-glucose 1-*P*-uridylyl transferase) and glucose-6P (PGM, phosphoglucomutase). Both products are precursors of trehalose-6-phosphate. As observed for ectoines metabolism, this correlation pattern of the trehalose metabolism module did not vary with salinity (Fig. 8).

The predicted modular distribution of correlated compatible solutes metabolism sets of reactions is very intriguing, and deserves experimental work to better understand *C. salexigens* osmoadaptive mechanisms, and especially ectoines production. Most of them were cyclic (not only the ectoine futile cycle), independent from other subsystems, and with no interdependence pattern variation in response to salinity. It is plausible that reactions included in each module respond to a similar regulation, and/or that a coordinated functioning of cyclic modules is necessary to adapt solute compatible metabolism, and therefore metabolic osmoadaptation, to external conditions. Our results are only an example (“the tip of the iceberg”) of the potential of the joint rational use of computational tools, together with experimental data, in order to explore metabolism in halophilic microorganisms.

## Conclusions

The information resulting from this work showed that the *i*FP764 model is a powerful tool to simulate and explore *C. salexigens* metabolism at different salinity conditions. This halophilic bacterium has one of the broadest salinity range of growth, and is considered as a model to study osmoadaptation. Notwithstanding the existing limitations in reconstructing and validating a *C. salexigens* specific network, the *i*FP764 model is one of the most complete and curated networks of halophilic bacteria, and accounts for all existing relevant experimental information. Thus, it may become an important tool to understand the metabolism of halophilic microorganisms, helping to drive the generation of new experimental hypotheses. In addition, *C. salexigens* is considered as a good cell factory for ectoines, whose production is salinity-dependent. Therefore, this model could be a robust and reliable tool to be used for the design of new *C. salexigens* strains optimized for the production of ectoines and other biotechnologically interesting compounds, avoiding the expenditure of resources.

## Additional files

**Additional file 1: Table S1.** Biomass composition at high salinity (´´BIO_H``). **Table S2.** Biomass composition at low salinity (´´BIO_L``); **Table S3.** Composition of M63 minimal medium and the exchange reactions formulated to simulate the uptake of metabolites from medium; **Table S4.** In silico (computational) constraints used for simulation at high or low salinity; Description of the bottom-up building and exhaustive manual refinement of the *C. salexigens* metabolic reconstruction *i*FP764; **Table S5.** The total number of dead ends metabolites found in the *i*FP764 model: root no-production metabolites and root no-consumption metabolites; **Table S6.** In silico prediction of utilization of various metabolites as carbon sources in *C. salexigens*; **Figure S1.** Selected histograms of possible flux values obtained in the first scenario by Monte Carlo sampling at low and high salinity relative to salinity-specific glucose consumption rate.

**Additional file 2.** Genome-scale metabolic reconstruction of *C. salexigens.* Details of *i*PF764 containing all genes associated-reactions and metabolites.

**Additional file 3: Table S7.** Essential genes at low and high salinity and their associated COGs.

## References

[1] Arahal DR, García MT, Vargas C, Cánovas D, Nieto JJ, Ventosa A (2001). *Chromohalobacter salexigens* sp. nov., a moderately halophilic species that includes *Halomonas elongata* DSM 3043 and ATCC 33174. Int J Syst Evol Microbiol.

[2] Vargas C, Argandoña M, Reina-Bueno M, Rodríguez-Moya J, Fernández-Aunión C, Nieto JJ (2008). Unravelling the adaptation responses to osmotic and temperature stress in *Chromohalobacter salexigens*, a bacterium with broad salinity tolerance. Saline Syst.

[3] Fallet C, Rohe P, Franco-Lara E (2010). Process optimization of the integrated synthesis and secretion of ectoine and hydroxyectoine under hyper/hypo-osmotic stress. Biotechnol Bioeng.

[4] Rodríguez-Moya J, Argandoña M, Iglesias-Guerra F, Nieto JJ, Vargas C (2013). Temperature- and salinity-decoupled overproduction of hydroxyectoine by *Chromohalobacter salexigens*. Appl Environ Microbiol.

[5] Argandoña M, Vargas C, Reina-Bueno M, Rodríguez-Moya J, Salvador M, Nieto JJ, Lorence A (2012). An extended suite of genetic tools for use in bacteria of the *Halomonadaceae*: an overview. Recombinant gene expression: reviews and protocols. Methods molecular biology.

[6] Copeland A, O’Connor K, Lucas S, Lapidus A, Berry KW, Detter JC, Del Rio TG, Hammon N, Dalin E, Tice H, Pitluck S, Bruce D, Goodwin L, Han C, Tapia R, Saunders E, Schmutz J, Brettin T, Larimer F, Land M, Hauser L (2011). Complete genome sequence of the halophilic and highly halotolerant *Chromohalobacter salexigens* type strain (1H11T). Stand Genomic Sci..

[7] Pastor JM, Bernal V, Salvador M, Argandoña M, Vargas C, Csonka L, Sevilla A, Iborra JL, Nieto JJ, Cánovas M (2013). Role of central metabolism in the osmoadaptation of the halophilic bacterium *Chromohalobacter salexigens*. J Biol Chem.

[8] O’Brien EJ, Monk JM, Palsson BO (2015). Using genome-scale models to predict biological capabilities. Cell.

[9] Lewis NE, Nagarajan H, Palsson BO (2012). Constraining the metabolic genotype-phenotype relationship using a phylogeny of in silico methods. Nat Rev Microbiol.

[10] Price ND, Reed JL, Palsson BO (2004). Genome scale models of microbian cells: evaluating the consequences of constraints. Nat Rev Microbiol.

[11] Papin JA, Price ND, Wiback SJ, Fell DA, Palsson BO (2003). Metabolic pathways in the post-genome era. Trends Biochem Sci.

[12] Lee KH, Park JH, Kim TY, Kim HU, Lee SY (2007). Systems metabolic engineering of *Escherichia coli* for l-threonine production. Mol Syst Biol.

[13] Pharkya P, Burgard AP, Maranas CD (2003). Exploring the overproduction of amino acids using the bilevel optimization framework OptKnock. Biotechnol Bioeng.

[14] Reed JL, Famili I, Thiele I, Palsson BO (2006). Towards multidimensional genome annotation. Nat Rev Genet.

[15] Altschul SF, Gish W, Miller W, Myers EW, Lipman DJ (1990). Basic local alignment search tool. J Mol Biol.

[16] Marchler-Bauer A, Panchenko AR, Shoemaker BA, Thiessen PA, Geer LY, Bryant SH (2002). CDD: a database of conserved domain alignments with links to domain three-dimensional structure. Nucleic Acids Res.

[17] Schultz J, Milpetz F, Bork P, Ponting CP (1998). SMART, a simple modular architecture research tool: identification of signaling domains. Proc Natl Acad Sci.

[18] Snel B, Lehmann G, Bork P, Huynen MA (2000). STRING: a web-server to retrieve and display the repeatedly occurring neighborhood of a gene. Nucleic Acids Res.

[19] Despalins A, Marsit S, Oberto J (2011). Absynte: a web tool to analyze the evolution of orthologous archaeal and bacterial gene clusters. Bioinformatics.

[20] Thompson JD, Higgins DG, Gibson TJ (1990). CLUSTAL W: improving the sensitivity of progressive multiple sequence alignment through sequence weighting, position-specific gap penalties and weight matrix choice. Nucleic Acids Res..

[21] Tamura K, Stecher G, Peterson D, Filipski A, Kumar S (2013). MEGA6: molecular evolutionary genetics analysis version 6.0. Mol Biol Evol.

[22] Schomburg I, Chang A, Schomburg D (2002). BRENDA, enzyme data and metabolic information. Nucleic Acids Res..

[23] Thiele I, Palsson BO (2010). A protocol for generating a high-quality genome-scale metabolic reconstruction. Nat Protoc.

[24] Vargas C, Kallimanis A, Koukkou AI, Calderon MI, Canovas D, Iglesias-Guerra F, Drainas C, Ventosa A, Nieto JJ (2005). Contribution of chemical changes in membrane lipids to the osmoadaptation of the halophilic bacterium *Chromohalobacter salexigens*. Syst Appl Microbiol.

[25] Orth JD, Conrad TM, Na J, Lerman JA, Nam H, Feist AM, Palsson BO (2011). A comprehensive genome-scale reconstruction of *Escherichia coli* metabolism. Mol Syst Biol..

[26] Neidhardt FC, Van Bogelen RA, Neidhardt FC, Ingraham JL, Low KB, Magasanik B, Schaechter M, Umbarger HE (1987). Heat shock response. *Escherichia coli* and *Salmonella typhimurium*; cellular and molecular biology. American society for microbiology.

[27] Cayley S, Record MT (2003). Roles of cytoplasmic osmolytes, water, and crowding in the response of *Escherichia coli* to osmotic stress: biophysical basis of osmoprotection by glycine betaine. Biochemistry.

[28] Feist AM, Henry CS, Reed JL, Krummenacker M, Joyce AR, Karp PD, Broadbelt LJ, Hatzimanikatis V, Palsson BO (2007). A genome-scale metabolic reconstruction for *Escherichia coli* K-12 MG1655 that accounts for 1260 ORFs and thermodynamic information. Mol Syst Biol..

[29] Feist AM, Palsson BO (2010). The biomass objective function. Curr Opin Microbiol.

[30] Reed JL, Patel TR, Chen KH, Joyce AR, Applebee MK, Herring CD, Bui OT, Knight EM, Fong SS, Palsson BO (2006). Systems approach to refining genome annotation. Proc Natl Acad Sci USA..

[31] Becker SA, Feist AM, Mo ML, Hannum G, Palsson BO, Herrgard MJ (2007). Quantitative prediction of cellular metabolism with constraint-based models: the COBRA Toolbox. Nat Protoc.

[32] Csonka LN (1982). A third l-proline permease in *Salmonella typhymurium* which functions in media of elevated osmotic strength. J Bacteriol.

[33] Kumar SV, Dasika MS, Maranas CD (2007). Optimization based automated curation of metabolic reconstructions. BMC Bioinform.

[34] Orth JD, Palsson BO (2010). Systematizing the generation of missing metabolic knowledge. Biotechnol Bioeng.

[35] Wiback SJ, Famili I, Greengerg HJ, Palsson BO (2004). Monte Carlo sampling can be used to determine the size and shape of the steady-state flux space. J Theor Biol.

[36] Lawrence J (1991). Polytope volume computation. Math Comput..

[37] Bartell JA, Blazier AS, Yen P, Thøgersen JC, Jelsbak L, Goldberg JB, Papin JA (2017). Reconstruction of the metabolic network of *Pseudomonas aeruginosa* to interrogate virulence factor synthesis. Nat Commun..

[38] Oh Y-K, Palsson BØ, Park SM, Schilling CH, Mahadevan R (2007). Genome-scale reconstruction of metabolic network in *Bacillus subtilis* based on high-throughput phenotyping and gene essentiality data. J Biol Chem.

[39] McAnulty MJ, Yen JY, Freedman BG, Senger RS (2012). Genome-scale modeling using flux ratio constraints to enable metabolic engineering of clostridial metabolism in silico. BMC Syst Biol.

[40] Diken E, Ozer T, Arikan M, Emrence Z, Oner ET, Ustek D, Arga KY (2015). Genomic analysis reveals the biotechnological and industrial potential of levan producing halophilic extremophile, *Halomonas smyrnensis* AAD6T. SpringerPlus.

[41] Tatusov RL, Koonin EV, Lipman DJ (1997). A genomic perspective on protein families. Science.

[42] Ates O, Oner ET, Arga KY (2011). Genome-scale reconstruction of metabolic network for a halophilic extremophile, *Chromohalobacter salexigens* *DSM3043*. BMC Syst Biol.

[43] Ponce-de-León M, Montero F, Peretó J (2013). Solving gap metabolites and blocked reactions in genome-scale models: application to the metabolic network of *Blattabacterium cuenoti*. BMC Syst Biol.

[44] Larocque M, Chénard T, Najmanovich R (2014). A curated *C. difficile* strain 630 metabolic network: prediction of essential targets and inhibitors. BMC Syst Biol.

[45] Schatschneider S, Persicke M, Watt SA, Hublik G, Puhler A, Niehaus K, Vorholter FJ (2013). Establishment, in silico analysis, and experimental verification of a large-scale metabolic network of the xanthan producing *Xanthomonas campestris pv. campestris* strain B100. J Biotechnol..

[46] Kitano H (2007). Towards a theory of biological robustness. Mol Syst Biol..

[47] Ye YN, Hua ZG, Huang J, Rao N (2013). Guo FB1. CEG: a database of essential gene clusters. BMC Genom..

[48] Oren A (1999). Bioenergetic aspects of halophilism. Microbiol Mol Biol Rev.

[49] Puchałka J, Oberhardt MA, Godinho M, Bielecka A, Regenhardt D, Timmis KN, Papin JA, Martins dos Santos VA (2008). Genome-scale reconstruction and analysis of the *Pseudomonas putida* KT2440 metabolic network facilitates applications in biotechnology. PLoS Comput Biol.

[50] Pfeiffer T, Schuster S (2005). Game-theoretical approaches to studying the evolution of biochemical systems. Trends Biochem Sci.

[51] Schuster S, Pfeiffer T, Fell DA (2008). Is maximization of molar yield in metabolic networks favored by evolution?. J Theor Biol.

[52] Metris A, George S, Baranyi S (2012). Modelling osmotic stress by Flux Balance Analysis at the genomic scale. Int J Food Microbiol.

[53] Covert MW, Palsson BO (2002). Transcriptional regulation in constraints-based metabolic models of *Escherichia coli*. J Biol Chem.

[54] Papin JA, Reed JL, Palsson BO (2004). Hierarchical thinking in network biology: the unbiased modularization of biochemical networks. Trends Biochem Sci.

[55] Nikkel PI, Chavarría M, Fuhrer T, Sauer U, de Lorenzo V (2015). *Pseudomonas putida* KT2440 strain metabolizes glucose through a cycle formed by enzymes of the Entner–Doudoroff, Embden–Meyerhof–Parnas and Pentose phosphate pathways. J Biol Chem.

[56] Schwibbert K, Marin-Sanguino A, Bagyan I, Heidrich G, Lentzen G, Seitz H, Rampp M, Schuster SC, Klenk HP, Pfeiffer F, Oesterhelt D, Kunte HJ (2011). A blueprint of ectoine metabolism from the genome of the industrial producer *Halomonas elongata* DSM 2581T. Environ Microbiol.

