# Supplementary material for: Insights into metabolic osmoadaptation of the ectoines-producer bacterium Chromohalobacter salexigens through a high-quality genome scale metabolic model
Source: Microb Cell Fact. 2018 Jan 9;17:2. doi: 10.1186/s12934-017-0852-0 (PMC5759318; doi:10.1186/s12934-017-0852-0)
Supplement: Supplementary file 1 — Additional file 1: Table S1. Biomass composition at high salinity (´´BIO_H``). Table S2. Biomass composition at low salinity (´´BIO_L``); Table S3. Composition of M63 minimal medium and the exchange reactions formulated to simulate the uptake of metabolites from medium; Table S4. In silico (computational) constraints used for simulation at high or low salinity; Description of the bottom-up building and exhaustive manual refinement of the C. salexigens metabolic reconstruction iFP764; Table S5. The total number of dead ends metabolites found in the iFP764 model: root no-production metabolites and root no-consumption metabolites; Table S6. In silico prediction of utilization of various metabolites as carbon sources in C. salexigens; Figure S1. Selected histograms of possible flux values obtained in the first scenario by Monte Carlo sampling at low and high salinity relative to salinity-specific glucose consumption rate. [file 12934_2017_852_MOESM1_ESM.docx]

**Insights into metabolic osmoadaptation of the ectoines-producer bacterium *Chromohalobacter salexigens* through a high-quality genome scale metabolic model**

Francine Piubeli, Manuel Salvador, Montserrat Argandoña**,** Joaquín J. Nieto, Vicente Bernal, Jose M. Pastor, Manuel Cánovas, and Carmen Vargas

**Additional Material:**

*Contents:*

1. **Table S1:** Biomass composition at high salinity (´´BIO_H).
2. **Table S2:** Biomass composition at low salinity (´´BIO_L``).
3. **Table S3:** Composition of M63 minimal medium and the exchange reactions formulated to simulate the uptake of metabolites from medium.
4. **Table S4:** *In silico* (computational) constrains used for simulation at high or low salinity.
5. Description of the bottom-up building and exhaustive manual refinement of the *C. salexigens* metabolic reconstruction *i*FP764
6. **Table S5:** The total number of dead ends metabolites found in the *i*FP764 model: root no-production metabolites and root no-consumption metabolites.
7. **Table S6:** *In silico* prediction of utilization of various metabolites as carbon sources in *C. salexigens*
8. **Figure S1.** Selected histograms of possible flux values obtained in the first scenario by Monte Carlo sampling at low and high salinity relative to salinity-specific glucose consumption rate.
9. References

**Table S1. Biomass composition at high salinity (´´BIO_H``).** All the experiments related to obtain the values for the ´´BIO_H`` was carried out at 2.5 M of NaCl.

| Macromolecule | overall wt% | composition (molar fraction) | mmol/gDW (Calc.) | metabolite |
| --- | --- | --- | --- | --- |
| Protein | 0,205 | 0,115 | 0,215287 | ala-L |
|  |  | 0,075 | 0,139700 | arg-L |
|  |  | 0,024 | 0,045647 | asn-L |
|  |  | 0,061 | 0,114285 | asp-L |
|  |  | 0,010 | 0,017859 | cys-L |
|  |  | 0,037 | 0,070118 | gln-L |
|  |  | 0,062 | 0,116021 | glu-L |
|  |  | 0,081 | 0,151978 | gly |
|  |  | 0,026 | 0,047708 | his-L |
|  |  | 0,046 | 0,085461 | ile-L |
|  |  | 0,113 | 0,210541 | leu-L |
|  |  | 0,025 | 0,046844 | lys-L |
|  |  | 0,025 | 0,046992 | met-L |
|  |  | 0,034 | 0,064392 | phe-L |
|  |  | 0,049 | 0,091691 | pro-L |
|  |  | 0,054 | 0,100388 | ser-L |
|  |  | 0,052 | 0,097638 | thr-L |
|  |  | 0,015 | 0,027963 | trp-L |
|  |  | 0,024 | 0,044776 | tyr-L |
|  |  | 0,072 | 0,134842 | val-L |
| DNA | 0,031 | 0,180 | 0,018178 | datp |
|  |  | 0,318 | 0,032029 | dctp |
|  |  | 0,321 | 0,032414 | dgtp |
|  |  | 0,181 | 0,018210 | dttp |
| RNA | 0,21 | 0,242 | 0,152315 | ctp |
|  |  | 0,375 | 0,236514 | gtp |
|  |  | 0,179 | 0,113148 | utp |
|  |  | 0,231 | 0,145440 | atp** |
| glycogen | 0,025 | 1 | 0,374000 | glycogen |
| murein | 0,025 | 0,4 | 0,028000 | murein5p5p |
| LPS | 0,034 | 1 | 0,019456 | kdo2lipid4 |
| lipid | 0,093 | 0,1800 | 0,027172 | pe160 |
|  |  | 0,0408 | 0,006159 | pe161 |
|  |  | 0,1498 | 0,022607 | pe181 |
|  |  | 0,0833 | 0,012567 | pg160 |
|  |  | 0,0189 | 0,002848 | pg161 |
|  |  | 0,0693 | 0,010456 | pg181 |
| inorganic ions | 0,01 | 0,7143 | 0,169185 | k |
|  |  | 0,0476 | 0,011279 | nh4 |
|  |  | 0,0317 | 0,007519 | mg2 |
|  |  | 0,0190 | 0,004512 | ca2 |
|  |  | 0,0286 | 0,006767 | fe2 |
|  |  | 0,0286 | 0,006767 | fe3 |
|  |  | 0,0127 | 0,003008 | cu2 |
|  |  | 0,0127 | 0,003008 | mn2 |
|  |  | 0,0127 | 0,003008 | mobd |
|  |  | 0,0127 | 0,003008 | cobalt2 |
|  |  | 0,0127 | 0,003008 | zn2 |
|  |  | 0,0190 | 0,004512 | cl |
|  |  | 0,0159 | 0,003760 | so4 |
|  |  | 0,0159 | 0,003760 | pi |
|  |  |  | 0,000279 | accoa |
|  |  |  | 0,000168 | coa |
|  |  |  | 0,000098 | succoa |
|  |  |  | 0,001787 | nad |
|  |  |  | 0,000045 | nadh |
|  |  |  | 0,000112 | nadp |
|  |  |  | 0,665011 | ect-L |
|  |  |  | 0,377131 | hdect |
|  |  |  | 0,000335 | nadph |
|  |  |  | 0,000223 | fad |
|  |  |  | 0,000223 | 5mthf |
|  |  |  | 0,000223 | hemeO |
|  |  |  | 0,000223 | sheme |
|  |  | 1 | 59,810000 | atp |
|  |  | 1 | 59,810000 | h2o |
|  |  |  | 59,955440 | atp |
|  |  |  | 57,939868 | h2o |

**Table S2: Biomass composition at low salinity (´´BIO_L``).** All the experiments related to obtain the values for the ´´BIO_L`` was carried out at 0.6 M of NaCl.

| Macromolecule | overall wt% | composition (molar fraction) | mmol/gDW (Calc.) | metabolite |
| --- | --- | --- | --- | --- |
| Protein | 0,35 | 0,115 | 0,367563 | ala-L |
|  |  | 0,075 | 0,238512 | arg-L |
|  |  | 0,024 | 0,077933 | asn-L |
|  |  | 0,061 | 0,195121 | asp-L |
|  |  | 0,010 | 0,030492 | cys-L |
|  |  | 0,037 | 0,119714 | gln-L |
|  |  | 0,062 | 0,198085 | glu-L |
|  |  | 0,081 | 0,259475 | gly |
|  |  | 0,026 | 0,081452 | his-L |
|  |  | 0,046 | 0,145908 | ile-L |
|  |  | 0,113 | 0,359460 | leu-L |
|  |  | 0,025 | 0,079978 | lys-L |
|  |  | 0,025 | 0,080230 | met-L |
|  |  | 0,034 | 0,109938 | phe-L |
|  |  | 0,049 | 0,156546 | pro-L |
|  |  | 0,054 | 0,171394 | ser-L |
|  |  | 0,052 | 0,166699 | thr-L |
|  |  | 0,015 | 0,047741 | trp-L |
|  |  | 0,024 | 0,076447 | tyr-L |
|  |  | 0,072 | 0,230218 | val-L |
| DNA | 0,031 | 0,180 | 0,018178 | datp |
|  |  | 0,318 | 0,032029 | dctp |
|  |  | 0,321 | 0,032414 | dgtp |
|  |  | 0,181 | 0,018210 | dttp |
| RNA | 0,21 | 0,242 | 0,152315 | ctp |
|  |  | 0,375 | 0,236514 | gtp |
|  |  | 0,179 | 0,113148 | utp |
|  |  | 0,231 | 0,145440 | atp** |
| glycogen | 0,025 | 1 | 0,218000 | glycogen |
| murein | 0,025 | 0,4 | 0,028000 | murein5p5p |
| LPS | 0,034 | 1 | 0,019456 | kdo2lipid4 |
| lipid | 0,093 | 0,2100 | 0,026820 | pe160 |
|  |  | 0,0640 | 0,008169 | pe161 |
|  |  | 0,2377 | 0,030362 | pe181 |
|  |  | 0,0976 | 0,012462 | pg160 |
|  |  | 0,0297 | 0,003796 | pg161 |
|  |  | 0,1105 | 0,014108 | pg181 |
| inorganic ions | 0,01 | 0,7143 | 0,169185 | k |
|  |  | 0,0476 | 0,011279 | nh4 |
|  |  | 0,0317 | 0,007519 | mg2 |
|  |  | 0,0190 | 0,004512 | ca2 |
|  |  | 0,0286 | 0,006767 | fe2 |
|  |  | 0,0286 | 0,006767 | fe3 |
|  |  | 0,0127 | 0,003008 | cu2 |
|  |  | 0,0127 | 0,003008 | mn2 |
|  |  | 0,0127 | 0,003008 | mobd |
|  |  | 0,0127 | 0,003008 | cobalt2 |
|  |  | 0,0127 | 0,003008 | zn2 |
|  |  | 0,0190 | 0,004512 | cl |
|  |  | 0,0159 | 0,003760 | so4 |
|  |  | 0,0159 | 0,003760 | pi |
|  |  |  | 0,000279 | accoa |
|  |  |  | 0,000168 | coa |
|  |  |  | 0,000098 | succoa |
|  |  |  | 0,001787 | nad |
|  |  |  | 0,000045 | nadh |
|  |  |  | 0,000112 | nadp |
|  |  |  | 0,000335 | nadph |
|  |  |  | 0,000223 | fad |
|  |  |  | 0,000223 | 5mthf |
|  |  |  | 0,000223 | hemeO |
|  |  |  | 0,242500 | ect-L |
|  |  |  | 0,009393 | hdect |
|  |  |  | 0,000223 | sheme |
|  |  | 1 | 59,810000 | atp |
|  |  | 1 | 59,810000 | h2o |
|  |  |  | 59,955440 | atp |
|  |  |  | 57,939868 | h2o |

**Table S3. Composition of M63 minimal medium and the exchange reactions formulated to simulate the uptake of metabolites from medium.**

| **Minimum medium composition** | **Exchange reaction ID** |
| --- | --- |
| KOH | K[e] |
| KH2PO4 | K[e]; pi[e] |
| (NH4)2SO4 | NH4[e]; SO4[e] |
| MgSO4 | Mg2[e]; SO4[e] |
| FeSO4.7H2O | Fe2[e]; SO4[e] |

**Table S4.** ***In silico* (computational) constrains used for simulation at high or low salinity.** The consumption (positive values) and excretion (negative values) rates were obtained from results described in Pastor et al., 2013 and are expressed in mmol.(g_cdw_h)^-1^ and used as constraints to simulate at low and high salinity conditions.

| **Metabolite Name** | **Metabolite abbreviation** | **Exchange Reaction** | **Constraints on the high salinity simulation (2.5 M)**  **(mmol.(g_cdw_h)^-1^)** | **Constraints on the low salinity simulation (0.6 M) (mmol.(g_cdw_h)^-1^)** |
| --- | --- | --- | --- | --- |
| Glucose | glc-D[c] | glc-D[e] <=> | -2.1 | -14.28 |
| Pyruvate | pyr[c] | pyr[c] -> | 0.30 | 2.25 |
| Acetate | ac[c] | ac[c] -> | 0.02 | 0.43 |
| Ammonium  Gluconate | NH4[c]  glcn[c] | nh4[e] <=>  glcn[p] -> | -2.48  0.17 | -3.73  0.66 |

***Bottom-up building and exhaustive manual refinement of the reconstruction***

A robust core metabolic model of *C. salexigens* was rationally constructed, which included pathways for the transport, synthesis and degradation of compatible solutes, as well as central C and N metabolism, biosynthesis of cell wall and membrane lipids, synthesis of cofactors and vitamins, uptake of ions, and biomass constituents.

*Synthesis of compatible solutes*

As *C. salexigens* is a halophilic microorganism, the complete routes for the synthesis and degradation of its main compatible solutes used for osmoadaptation were included in the reconstruction in order to ensure the quality of simulations. For ectoines, several routes were incorporated, such as those for ectoine degradation (encoded by *doeABCD*) [1], ectoine synthesis (encoded by *ectABC*) [2], ectoine hydroxylase (encoded by *ectD*) [3] and the recently described alternative route for hydroxyectoine degradation catalysed by the EutB and EutC enzymes [4] (see Figure 2A).

Pathways for the synthesis and degradation of glycine-betaine and trehalose were also included in the reconstruction. The glycine-betaine catabolism route, not completely annotated in the genome, was filled up by adding the gene *csal0990*, responsible for the demethylation of dimethylglycine to sarcosine. Trehalose can be synthesized from glucose using the metabolic pathway catalysed by OtsA and OtsB enzymes [5]. Trehalose can also be used as a carbon source [6]. The genes *csal0235* (TreF), suggested by Reina-Bueno and co-workerss to be responsible for the degradation of trehalose [5], as well the synthesis genes (*otsAB*) and their associated reactions, were also included in the model.

*Central metabolism*

*C. salexigens* uses the Entner-Doudoroff pathway for glucose catabolism, rather than the standard glycolytic pathway and anaplerotic activity is high to replenish the TCA cycle with the intermediaries withdrawn for ectoines biosynthesis [7]. Consequently, metabolism in this organism has to be adapted to support this biosynthetic route. A special effort was made to revise and correctly include all the central carbon metabolism routes re-annotated by Pastor and co-workers [7]. These include the pathways for glucose assimilation through the periplasmic and cytoplasmic variants of the of Entner-Doudoroff pathway.

The fructose 6-phosphofructokinase, present in the previous metabolic reconstruction of *Chromohalobacter salexigens* [8], was not included. The reason for this was the lack of the enzymatic activity [7], and the absence of a *bona fide* gene encoding this protein in the *C. salexigens* genome. The acetate and pyruvate routes were also revised due to their importance on the overflow metabolism in *C. salexigens*. In this way, the pyruvate oxidase (PoxB) and the AMP-forming acetyl-coenzyme synthetase (Acs) reactions were included, among others.

*Amino acid metabolism*

Regarding nitrogen metabolism, genes for the L-arginine, L-methionine and L-histidine biosynthetic pathways were not completely annotated in *C. salexigens* genome. Thus, their routes were filled and included in the reconstruction. Interestingly, these routes are probably involved in *C. salexigens* metabolic osmoadaptation, as they are differentially expressed at different salinity conditions (data not show). The *i*FP764 reconstruction contained the complete pathways for the synthesis of all amino acids.

*Biosynthesis of cell wall and membrane components*

Additionally to accumulating compatible solutes, halophilic microorganisms cope with osmotic stress by adapting their membrane lipid composition in response to salinity. Thus, all routes for the biosynthesis of lipopolysaccharide, peptidoglycan (cell wall), and membrane phospholipids were exhaustively refined based on previous works [9], transcriptomic data (data not show), and published metabolic reconstructions of gram negative bacteria [10,11,12].

*Other pathways: synthesis of cofactors and vitamins and biomass constituents.*

Regarding the metabolism of cofactors and vitamins, *C. salexigens* genome included pathways for the synthesis of thiamine, porphyrin and the hemo group, riboflavin, pyridoxal phosphate, lipoic acid and folate. All these pathways were refined and included in the model. In addition, the routes for the synthesis of biotin and ubiquinone, whose genes were found differentially expressed in our trancriptomic analysis (not shown), were added into the reconstruction.

Finally, all the pathways for the synthesis of the biomass components were incorporated, gaps were filled, and connectivity analyses were performed to complete the reconstruction.

**Table S5.** The total number of dead ends metabolites found in the *i*FP764 model: root no-production metabolites and root no-consumption metabolites.

| **Root no-production metabolites** | **Root no-consumption metabolites** |
| --- | --- |
| 2pglyc[c] | 2amsa[c] |
| 3sala[c] | 2amsa[c] |
| 4ahmmp[c] | 4hthr[c] |
| alltt[c] | 5mtr[c] |
| cph4[c] | acetol[c] |
| cu[c] | acgam[c] |
| dxyl[c] | acmum6p[c] |
| fru[c] | alatrna[c] |
| man[c] | argtrna[c] |
| mi1p-D[c] | asptrna[c] |
| o2s[c] | athtp[c] |
| suchms[c] | bmocogdp[c] |
| trnaala[c] | btamp[c] |
| trnaarg[c] | bwcogdp[c] |
| trnaasp[c] | cpe160[c] |
| trnahis[c] | cpe180[c] |
| trnaile[c] | cpg160[c] |
| trnaleu[c] | cpg180[c] |
| trnamet[c] | dca[e] |
| trnaphe[c] | ddca[e] |
| trnapro[c] | dhmptp[c] |
| trnasecys[c] | etha[c] |
| trnaser[c] | fe3dcit[e] |
| trnathr[c] | fmettrna[c] |
| trnatrp[c] | fruur[c] |
| trnaval[c] | gdpmann[c] |
| udcpp[p] | h2[c] |
|  | h2[p] |
|  | h2o2[p] |
|  | hhlipa[c] |
|  | histrna[c] |
|  | iletrna[c] |
|  | inost[c] |
|  | leutrna[c] |
|  | lipopb[c] |
|  | malt[e] |
|  | malthx[e] |
|  | maltpt[e] |
|  | malttr[e] |
|  | maltttr[e] |
|  | mococdp[c] |
|  | mocogdp[c] |
|  | ocdca[e] |
|  | ocdcea[e] |
|  | octa[e] |
|  | pe120[c] |
|  | pe140[c] |
|  | pe141[c] |
|  | pe180[c] |
|  | pg120[c] |
|  | pg140[c] |
|  | pg141[c] |
|  | pg160[c] |
|  | pg180[c] |
|  | phetrna[c] |
|  | preq1[c] |
|  | protrna[c] |
|  | s17bp[c] |
|  | sertrna[c] |
|  | sertrna[sec][c] |
|  | spmd[c] |
|  | tagur[c] |
|  | thmnp[c] |
|  | thrtrna[c] |
|  | trptrna[c] |
|  | ttdca[e] |
|  | ttdcea[e] |
|  | um4p[c] |
|  | ump[e] |
|  | valtrna[c] |
|  | xyl[c] |

**Table S6. *In silico* prediction of utilization of various metabolites as carbon sources in *C. salexigens***

| **Metabolite name** | **Compound** | **Metabolite formula** | ***In silico* growth rate (mmolgDW-1h-1)** |
| --- | --- | --- | --- |
| 5-Dehydro-D-gluconate | 5dglcn[e] | C6H9O7 | 0.9464 |
| Acetaldehyde | acald[e] | C2H4O | 0.3474 |
| Acyl carrier protein | ACP[e] | C11H21N2O7PRS | - |
| O-Acetyl-L-serine | acser[e] | C5H9NO4 | - |
| Adenine | ade[e] | C5H5N5 | 0.1694 |
| 2-Oxoglutarate | akg[e] | C5H4O5 | 0.6977 |
| D-Alanyl-D-alanine | alaala[e] | C6H12N2O3 | 1.0287 |
| Allantoin | alltn[e] | C4H6N4O3 | - |
| N-Acetyl-D-glucosamine(anhydrous)N-Acetylmuramic acid | anhgm[e] | C19H29N2O12 | - |
| apoACP | apoACP | RHO | - |
| aerobactin minus Fe3 | arbtn[e] | C22H33N4O13 | - |
| Aerobactin | arbtn-fe3[e] | C22H33FeN4O13 | - |
| L-Aspartate | asp-L[e] | C4H6NO4 | 0.5100 |
| Biotin | btn[e] | C10H15N2O3S | - |
| Cys-Gly | cgly[e] | C5H10N2O3S | 0.1878 |
| Coprogen | cpgn[e] | C35H52N6O13Fe | - |
| Coprogen unloaded (no Fe(III)) | cpgn-un[e] | C35H52N6O13 | - |
| Cytosine | csn[e] | C4H5N3O | - |
| L-Cysteine | cys-L[e] | C3H7NO2S | - |
| Decanoate | dca[e] | C10H19O2 | 1.6940 |
| Dodecanoate (n-C12:0) | ddca[e] | C12H23O2 | 2.0612 |
| dGMP | dgmp[e] | C10H12N5O7P | 1.1989 |
| Deoxyguanosine | dgsn[e] | C10H13N5O4 | 1.1989 |
| Dihydroxyacetone | dha[e] | C3H6O3 | 0.5273 |
| dIMP | dimp[e] | C10H11N4O7P | 1.2866 |
| Deoxyinosine | din[e] | C10H12N4O4 | 1.2866 |
| Ethanolamine | etha[e] | C2H8NO | - |
| Formaldehyde | fald[e] | CH2O | 0.0779 |
| Fe(III)dicitrate | fe3dcit[e] | C12H10FeO14 | - |
| Fe(III)hydroxamate | fe3hox[e] | C9H18O6N3Fe | - |
| Fe(III)hydroxamate | fe3hox-un[e] | C9H18O6N3 | - |
| Ferrichrome | fecrm[e] | C27H42FeN9O12 | - |
| Ferrichrome minus Fe(III) | fecrm-un[e] | C27H42N9O12 | - |
| ferroxamine | feoxam[e] | C25H46FeN6O8 | - |
| ferroxamine minus Fe(3) | feoxam-un[e] | C25H46N6O8 | - |
| sn-Glycero-3-phosphocholine | g3pc[e] | C8H20NO6P | 0.6832 |
| sn-Glycero-3-phosphoethanolamine | g3pe[e] | C5H14NO6P | 0.6774 |
| Glycerophosphoglycerol | g3pg[e] | C6H14O8P | 1.3457 |
| sn-Glycero-3-phospho-1-inositol | g3pi[e] | C6H14O8P | 0.6770 |
| Glycerophosphoserine | g3ps[e] | C6H13NO8P | 1.1306 |
| D-Glucose 6-phosphate | g6p[e] | C6H11O9P | 1.1218 |
| D-Galactonate | galctn-D[e] | C6H11O7 | 0.9844 |
| D-Glucosamine 6-phosphate | gam6p[e] | C6H13NO8P | - |
| GDP | gdp[e] | C10H12N5O11P2 | - |
| D-Glucarate | glcr[e] | C6H8O8 | 0.7764 |
| Glycerol 2-phosphate | glyc2p[e] | C3H7O6P | 0.5908 |
| Glycerol 3-phosphate | glyc3p[e] | C3H7O6P | 0.6774 |
| Glycolate | glyclt[e] | C2H3O3 | 0.1641 |
| GMP | gmp[e] | C10H12N5O8P | 1.1618 |
| Guanosine | gsn[e] | C10H13N5O5 | 1.1618 |
| Reduced glutathione | gthrd[e] | C10H16N3O6S | 1.0265 |
| GTP | gtp[e] | C10H12N5O14P3 | - |
| L-Histidine | his-L[e] | C6H9N3O2 | - |
| L-Homoserine | hom-L[e] | C4H9NO3 | - |
| Hexanoate (n-C6:0) | hxa[e] | C6H11O2 | 0.9598 |
| L-Isoleucine | ile-L[e] | C6H13NO2 | - |
| IMP | imp[e] | C10H11N4O8P | 1.2492 |
| Indole | indole[e] | C8H7N | - |
| myo-Inositol | inost[e] | C6H12O6 | - |
| Inosine | ins[e] | C10H12N4O5 | 1.2492 |
| KDO(2)-lipid IV A | kdo2lipid4[e] | C84H148N2O37P2 | - |
| L-alanine-D-glutamate-meso-2,6-diaminoheptanedioate | LalaDgluMdap[e] | C15H25N4O8 | - |
| L-alanine-D-glutamate-meso-2,6-diaminoheptanedioate-D-alanine | LalaDgluMdapDala[e] | C18H30N5O9 | 0.4862 |
| L-Leucine | leu-L[e] | C6H13NO2 | - |
| cold adapted KDO(2)-lipid (A) | lipa_cold[e] | C114H202N2O39P2 | - |
| Maltohexaose | malthx[e] | C36H62O31 | 6.6594 |
| Maltopentaose | maltpt[e] | C30H52O26 | 5.5344 |
| Maltotriose | malttr[e] | C18H32O16 | 3.2843 |
| Maltotetraose | maltttr[e] | C24H42O21 | 4.4092 |
| D-Mannose 6-phosphate | man6p[e] | C6H11O9P | 1.1218 |
| octadecanoate (n-C18:0) | ocdca[e] | C18H35O2 | 3.1572 |
| octadecenoate | ocdcea[e] | C18H33O2 | 3.2443 |
| octanoate (n-C8:0) | octa[e] | C8H15O2 | 1.3376 |
| Orotate | orot[e] | C5H3N2O4 | - |
| L-Phenylalanine | phe-L[e] | C9H11NO2 | - |
| Propanal | ppal[e] | C3H6O | 0.5674 |
| L-Prolinylglycine | progly[e] | C7H12N2O3 | 1.0264 |
| Pyruvate | pyr[e] | C3H3O3 | 0.3974 |
| D-Tagatose 6-phosphate | tag6p-D[e] | C6H11O9P | 1.1615 |
| L-tartrate | tartr-L[e] | C4H4O6 | 0.4098 |
| Thymine | thym[e] | C5H6N2O2 | - |
| L-Tryptophan | trp-L[e] | C11H12N2O2 | - |
| tetradecanoate (n-C14:0) | ttdca[e] | C14H27O2 | 2.4276 |
| tetradecenoate (n-C14:1) | ttdcea[e] | C14H25O2 | 2.5217 |
| L-Tyrosine | tyr-L[e] | C9H11NO3 | - |
| UMP | ump[e] | C9H11N2O9P | - |
| Uracil | ura[e] | C4H4N2O2 | - |
| Xanthine | xan[e] | C5H4N4O2 | 0.0954 |
| Xanthosine 5'-phosphate | xmp[e] | C10H11N4O9P | 1.0960 |
| Xanthosine | xtsn[e] | C10H12N4O6 | 1.0960 |


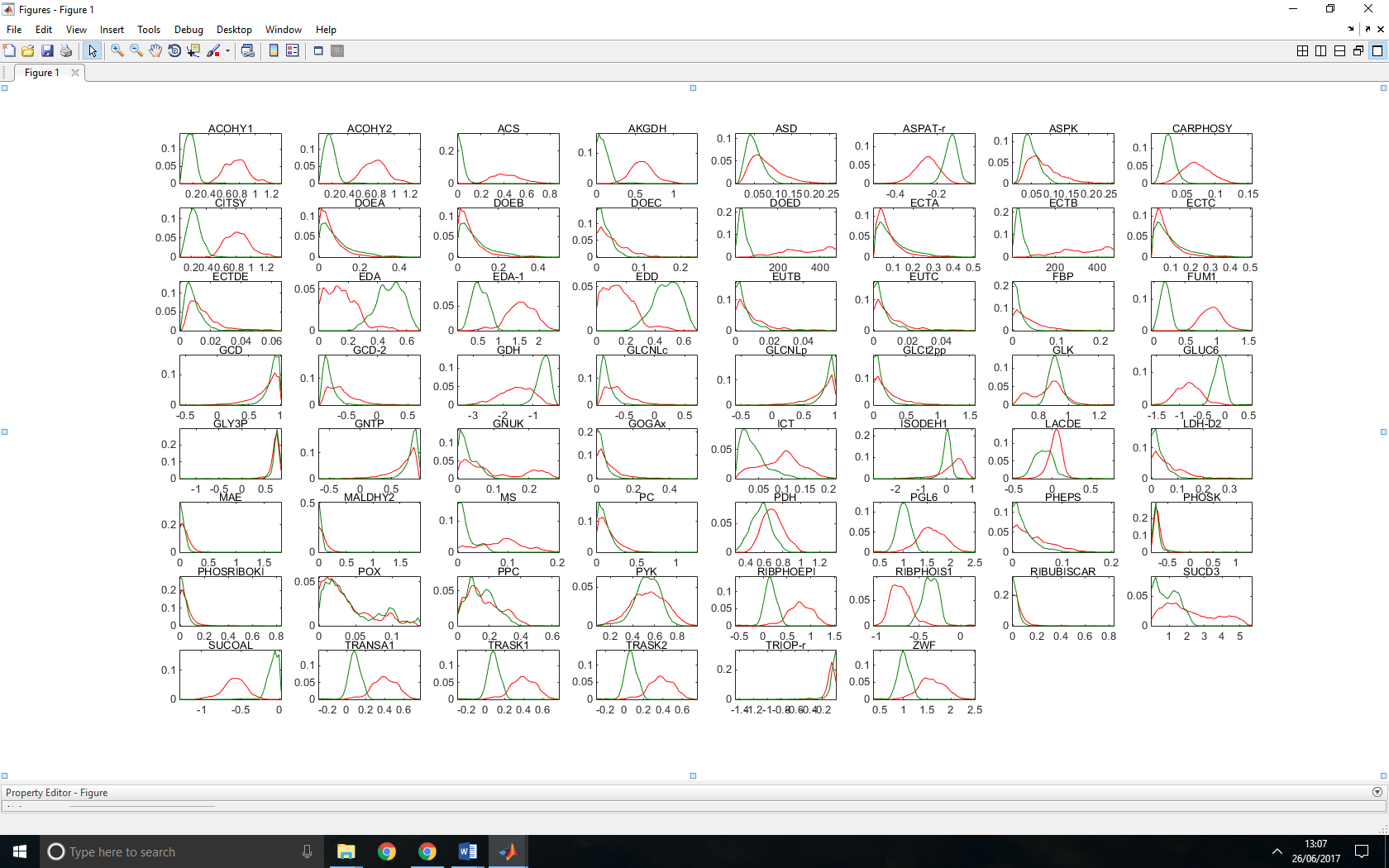


**Figure S1. Selected histograms of possible flux values obtained in the first scenario by Monte Carlo sampling at low and high salinity relative to salinity-specific glucose consumption rate**. Each histogram show one-dimensional information on its x axis, in terms of the extent of possible values for that particular flux. The y axis represents the “size” of space in the other r–1 dimensions resulting from slicing the metabolic solution space along a specific value of the flux through the indicated reaction. The red lines represent the possible fluxes obtained at high salinity and the green line, to the fluxes at low salinity.

**REFERENCES**

1. Schwibbert K, Marin-Sanguino A, Bagyan I, [Heidrich G](https://www.ncbi.nlm.nih.gov/pubmed/?term=Heidrich%20G%5BAuthor%5D&cauthor=true&cauthor_uid=20849449), [Lentzen G](https://www.ncbi.nlm.nih.gov/pubmed/?term=Lentzen%20G%5BAuthor%5D&cauthor=true&cauthor_uid=20849449), [Seitz H](https://www.ncbi.nlm.nih.gov/pubmed/?term=Seitz%20H%5BAuthor%5D&cauthor=true&cauthor_uid=20849449), [Rampp M](https://www.ncbi.nlm.nih.gov/pubmed/?term=Rampp%20M%5BAuthor%5D&cauthor=true&cauthor_uid=20849449), [Schuster SC](https://www.ncbi.nlm.nih.gov/pubmed/?term=Schuster%20SC%5BAuthor%5D&cauthor=true&cauthor_uid=20849449), [Klenk HP](https://www.ncbi.nlm.nih.gov/pubmed/?term=Klenk%20HP%5BAuthor%5D&cauthor=true&cauthor_uid=20849449), [Pfeiffer F](https://www.ncbi.nlm.nih.gov/pubmed/?term=Pfeiffer%20F%5BAuthor%5D&cauthor=true&cauthor_uid=20849449), [Oesterhelt D](https://www.ncbi.nlm.nih.gov/pubmed/?term=Oesterhelt%20D%5BAuthor%5D&cauthor=true&cauthor_uid=20849449), [Kunte HJ](https://www.ncbi.nlm.nih.gov/pubmed/?term=Kunte%20HJ%5BAuthor%5D&cauthor=true&cauthor_uid=20849449). A blueprint of ectoine metabolism from the genome of the industrial producer *Halomonas elongate* DSM 2581T. [Environ Microbiol.](https://www.ncbi.nlm.nih.gov/pubmed/20849449) 2011;13(8):1973–94.
2. Cánovas D, C Vargas, MI Calderón, A Ventosa, JJ Nieto. Characterization of the genes for the biosynthesis of the compatible solute ectoine in the moderately halophilic bacterium *Halomonas elongata* DSM 3043. Syst. Appl. Microbiol. 1998;21:487–97.
3. García-Estepa R, Argandoña M, Reina-Bueno M, C. Nieves, Inglesias-Guerra F, Nieto JJ, Vargas C. The *ectD* Gene, Which is involved in the synthesis of the compatible solute hydroxyectoine, is essential for thermoprotection of the halophilic bacterium *Chromohalobacter salexigens.* [J Bacteriol](https://www.ncbi.nlm.nih.gov/pmc/articles/PMC1482885/). 2006;188(11):3774–84.
4. [Schulz A](https://www.ncbi.nlm.nih.gov/pubmed/?term=Schulz%20A%5BAuthor%5D&cauthor=true&cauthor_uid=27318028), [Stöveken N](https://www.ncbi.nlm.nih.gov/pubmed/?term=St%C3%B6veken%20N%5BAuthor%5D&cauthor=true&cauthor_uid=27318028), [Binzen IM](https://www.ncbi.nlm.nih.gov/pubmed/?term=Binzen%20IM%5BAuthor%5D&cauthor=true&cauthor_uid=27318028), [Hoffmann T](https://www.ncbi.nlm.nih.gov/pubmed/?term=Hoffmann%20T%5BAuthor%5D&cauthor=true&cauthor_uid=27318028), [Heider J](https://www.ncbi.nlm.nih.gov/pubmed/?term=Heider%20J%5BAuthor%5D&cauthor=true&cauthor_uid=27318028), [Bremer E](https://www.ncbi.nlm.nih.gov/pubmed/?term=Bremer%20E%5BAuthor%5D&cauthor=true&cauthor_uid=27318028). Feeding on compatible solutes: A substrate-induced pathway for uptake and catabolism of ectoines and its genetic control by EnuR. [Environ Microbiol.](https://www.ncbi.nlm.nih.gov/pubmed/27318028) 2017;19(3):926–946.
5. Reina-Bueno M, Argandoña M, Salvador M, Rodríguez-Moya J, Iglesias-Guerra F, Csonka LN, Nieto JJ, Vargas C. Role of trehalose in salinity and temperature tolerance in the model halophilic bacterium *Chromohalobacter salexigens*. PLoS One. 2012;7(3):e33587.
6. Arahal DR, García MT, Vargas C, Cánovas D, Nieto JJ, Ventosa A. *Chromohalobacter salexigens* sp. nov., a moderately halophilic species that includes *Halomonas elongata* DSM 3043 and ATCC 33174. Int. J. Syst. Evol. Microbiol. 2001;51:1457-62.
7. Pastor JM, Bernal V, Salvador M, Argandoña M, Vargas C, Csonka L, Sevilla A, Iborra JL, Nieto JJ, Cánovas M. [Role of central metabolism in the osmoadaptation of the halophilic bacterium *Chromohalobacter salexigens*.](https://www.ncbi.nlm.nih.gov/pubmed/23615905) J Biol Chem.  2013;288(24):17769–81.
8. Ates O, Oner ET, Arga KY. Genome-scale reconstruction of metabolic network for a halophilic extremophile, *Chromohalobacter salexigens DSM 3043*. BMC Systems Biology 2011;5:12.
9. Vargas C, Kallimanis A, Koukkou AI, Calderon MI, Canovas D, Iglesias-Guerra F, Drainas C, Ventosa A, Nieto JJ. [Contribution of chemical changes in membrane lipids to the osmoadaptation of the halophilic bacterium *Chromohalobacter* *salexigens.*](https://www.ncbi.nlm.nih.gov/pubmed/16156114) Syst Appl Microbiol. 2005;28(7):571-81.
10. Feist AM, Henry CS, Reed JL,  [Krummenacker M](https://www.ncbi.nlm.nih.gov/pubmed/?term=Krummenacker%20M%5BAuthor%5D&cauthor=true&cauthor_uid=17593909), [Joyce AR](https://www.ncbi.nlm.nih.gov/pubmed/?term=Joyce%20AR%5BAuthor%5D&cauthor=true&cauthor_uid=17593909), [Karp PD](https://www.ncbi.nlm.nih.gov/pubmed/?term=Karp%20PD%5BAuthor%5D&cauthor=true&cauthor_uid=17593909), [Broadbelt LJ](https://www.ncbi.nlm.nih.gov/pubmed/?term=Broadbelt%20LJ%5BAuthor%5D&cauthor=true&cauthor_uid=17593909), [Hatzimanikatis V](https://www.ncbi.nlm.nih.gov/pubmed/?term=Hatzimanikatis%20V%5BAuthor%5D&cauthor=true&cauthor_uid=17593909), [Palsson BO](https://www.ncbi.nlm.nih.gov/pubmed/?term=Palsson%20B%C3%98%5BAuthor%5D&cauthor=true&cauthor_uid=17593909). A genome-scale metabolic reconstruction for Escherichia coli K-12 MG1655 that accounts for 1260 ORFs and thermodynamic information. [Mol Syst Biol.](https://www.ncbi.nlm.nih.gov/pubmed/17593909) 2007;3:121.
11. Orth JD, Conrad TM, Na J, [Lerman JA](https://www.ncbi.nlm.nih.gov/pubmed/?term=Lerman%20JA%5BAuthor%5D&cauthor=true&cauthor_uid=21988831), [Nam H](https://www.ncbi.nlm.nih.gov/pubmed/?term=Nam%20H%5BAuthor%5D&cauthor=true&cauthor_uid=21988831), [Feist AM](https://www.ncbi.nlm.nih.gov/pubmed/?term=Feist%20AM%5BAuthor%5D&cauthor=true&cauthor_uid=21988831), [Palsson BO](https://www.ncbi.nlm.nih.gov/pubmed/?term=Palsson%20B%C3%98%5BAuthor%5D&cauthor=true&cauthor_uid=21988831). A comprehensive genome-scale reconstruction of *Escherichia coli* metabolism. 2011. Molecular Systems Biology. 2011;7:535.
12. Nogales J, Palsson BO, Thiele I. A genome-scale metabolic reconstruction of *Pseudomonas putida* KT2440: iJN746 as a cell factory. BMC Systems Biology. 2008;2:79.
